# A chromosome-level phased genome enabling allele-level studies in sweet orange: a case study on citrus Huanglongbing tolerance

**DOI:** 10.1093/hr/uhac247

**Published:** 2022-11-03

**Authors:** Bo Wu, Qibin Yu, Zhanao Deng, Yongping Duan, Feng Luo, Frederick Gmitter Jr

**Affiliations:** School of Computing, Clemson University, 100 McAdams Hall, Clemson, SC 29643, USA; Department of Horticultural Sciences, Citrus Research and Education Center, University of Florida, IFAS, 700 Experiment Station Road, Lake Alfred, FL 33850, USA; Department of Environmental Horticulture, Gulf Coast Research and Education Center, University of Florida, IFAS, 14625 County Road 672, Wimauma, FL 33598, USA; USDA-ARS, U.S. Horticultural Research Laboratory, 2001 South Rock Road, Fort Pierce, FL 34945, USA; School of Computing, Clemson University, 100 McAdams Hall, Clemson, SC 29643, USA; Department of Horticultural Sciences, Citrus Research and Education Center, University of Florida, IFAS, 700 Experiment Station Road, Lake Alfred, FL 33850, USA

## Abstract

Sweet orange originated from the introgressive hybridizations of pummelo and mandarin resulting in a highly heterozygous genome. How alleles from the two species cooperate in shaping sweet orange phenotypes under distinct circumstances is unknown. Here, we assembled a chromosome-level phased diploid Valencia sweet orange (DVS) genome with over 99.999% base accuracy and 99.2% gene annotation BUSCO completeness. DVS enables allele-level studies for sweet orange and other hybrids between pummelo and mandarin. We first configured an allele-aware transcriptomic profiling pipeline and applied it to 740 sweet orange transcriptomes. On average, 32.5% of genes have a significantly biased allelic expression in the transcriptomes. Different cultivars, transgenic lineages, tissues, development stages, and disease status all impacted allelic expressions and resulted in diversified allelic expression patterns in sweet orange, but particularly citrus Huanglongbing (HLB) shifted the allelic expression of hundreds of genes in leaves and calyx abscission zones. In addition, we detected allelic structural mutations in an HLB-tolerant mutant (T19) and a more sensitive mutant (T78) through long-read sequencing. The irradiation-induced structural mutations mostly involved double-strand breaks, while most spontaneous structural mutations were transposon insertions. In the mutants, most genes with significant allelic expression ratio alterations (≥1.5-fold) were directly affected by those structural mutations. In T19, alleles located at a translocated segment terminal were upregulated, including *CsDnaJ*, *CsHSP17.4B*, and *CsCEBPZ*. Their upregulation is inferred to keep phloem protein homeostasis under the stress from HLB and enable subsequent stress responses observed in T19. DVS will advance allelic level studies in citrus.

## Introduction

Sweet orange (*Citrus sinensis* L.) originated from complex hybridization processes involving mandarins (*Citrus reticulata* Blanco) and pummelos [*Citrus maxima* (Burm.) Merr.] [1, 2]. Several other citrus cultivar groups also arose from interspecific introgressive hybridization events [3], such as lemon (*Citrus limon* L.) and grapefruit (*Citrus paradisi* Macfad.). The primitive *Citrus* species contributing to the hybridizations generally diverged between 3 and 8 million years ago and vary substantially in genomes and phenotypes [3, 4]. Interspecific and introgression hybrids in citrus are phenotypically distinct from their parents. How the allelic genes from diverged species cooperate in shaping the phenotypes of the hybrids under different conditions remains largely unknown. Phased genome assemblies are fundamental in deciphering the allelic contributions to horticultural traits of sweet orange and other hybrids. Moreover, sweet orange is important for studying citrus horticultural traits among its diverse cultivars arising from somatic mutations [5, 6], making a high-quality reference genome highly desirable.

Due to the difficulty of assembling a highly heterozygous genome, the current best sweet orange genome assembly was from a di-haploid sweet orange (HSO) [1, 5]. With a haploid reference genome, both somatic mutation calling and gene expression quantification could be compromised in the highly divergent genomic regions in sweet orange. Later efforts to sequence diploid sweet oranges only generated haploid-sized assemblies with inferior qualities [2, 5]. The mapping-based phasing method partitioned the diploid sweet orange genome into 325 phased blocks and failed to provide a reference-level genome [5]. Here, by adjusting the assembly procedure according to the intra-genomic allelic variance level, we successfully assembled a chromosome-level phased Valencia sweet orange (DVS) genome with significantly improved K-mer completeness, base accuracy, and gene annotation completeness compared to HSO v4 [5]. DVS harbors a high allelic-variance level and enables allele-level studies for hybrids between pummelo and mandarin.

The relative genetic uniformity of sweet orange cultivars and their use in monoculture production make them vulnerable to disease epidemics. The citrus Huanglongbing (HLB) is a devastating disease presumably caused by *Candidatus* Liberibacter asiaticus (*C*Las) [7], which is a phloem-limited bacterium. All commercial sweet orange cultivars are susceptible to HLB, and the selection of HLB tolerant/resistant germplasm has been considered the ultimate solution to this devastating disease. Although no absolute HLB immunity has been found in natural citrus germplasm, different degrees of HLB tolerance and sensitivity have been observed [8–14]. In this study, we have selected an HLB-tolerant 22-year-old irradiation-induced Valencia orange mutant, under disease pressure since HLB was found in Florida in 2005. By taking advantage of DVS, we could reveal the molecular mechanisms underlying its high HLB tolerance at the allelic level.

## Results

### Phased Valencia sweet orange genome assembly

**Figure 1:**
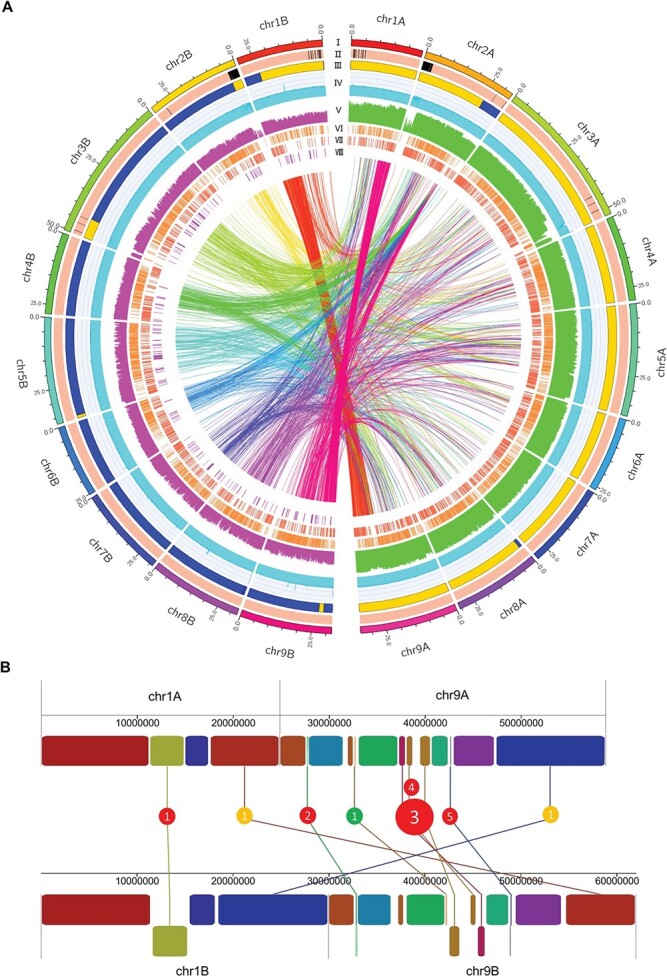
Characterization of the DVS genome and its intra-genomic variations. (A) Characteristics of the phased DVS assembly and whole-genome variation distribution. I. Ideogram of the DVS genome. The unit of the tick labels is million base pairs (Mb). (II) The phased (light red) and unphased region (black) in the assembly. (III) Distribution of the mandarin- (M, orange bands) and pummelo- (blue bands) origin chromosome regions. (IV) The binary logarithm of the depth of uniquely mapped CLR reads across DVS in 50 kb windows. Neighboring windows are overlapped by 20 kb. The vertical axis range from 0 to 16. (V) Histogram of the binary logarithm of ≤50 bp indel (purple) and SNV (green) counts in 100 kb non-overlapping continuous windows. The vertical axis range from 0 to 12. Tandem duplications (VI) and insertions (VII) in DVS_A (DVS_B) with DVS_B (DVS_A) as the reference, which could also be described as deletions in the other chromosome set. (VII) Inversions in DVS_B with DVS_A as the reference. The inner links show all inter-chromosomal translocations between DVS_A and DVS_B, which have been colored the same as the corresponding DVS_B chromosomes. (B) Seven large SVs on chr1A/B and chr9A/B. Rectangles with the same colors on chr1/9A and chr1/9B denote the orthologous blocks. The rectangles reversed in direction are shown in the bottom row on chr1B and chr9B. Red, yellow, and green circles denote INVs, inter-chromosomal translocations, and intra-chromosomal translocations, respectively.

We obtained 143.9 Gb (~ 420 ×) PacBio continuous long reads for an ordinary diploid Valencia sweet orange (DVS) genome. We first obtained a 607.6 Mb raw assembly using CANU, including 383 contigs with an N50 length of 15.4 Mb (Supplementary Table 1). Then we applied phased assembly to the collapsed and expanded regions (Supplementary Fig. 1A). Approximately 3.9 Mb runs of homozygosity (< 1‰ allelic variance) in 14 regions remain unphased in the final assembly, including 3.2 Mb at the 5′ end of chr2 (Fig. 1A). By resolving the repetitive units of long tandem-repeats (Supplementary Fig. 1B), we connected all filtered contigs into 18 pseudo-chromosomes totaling 598.6 Mb, which were assigned into two homologous chromosome sets DVS_A (chr1-9A, primarily mandarin-origin) and DVS_B (chr1-9B, primarily pummelo-origin) (Fig. 1A). An average hamming error rate of 0.18% was observed across the genome, and no switch errors were detected in the interspecific heterozygous regions.

The DVS assembly was estimated with 98.5% (K-mer) or 98.7% (BUSCO) completeness and an average error rate of 8.8E-6 (QV = 50.6). Its base error rate is 46.8-fold lower, and its K-mer-based completeness is 25.6% higher than HSO (if not specified, HSO denotes HSO v4) (Supplementary Table 2 and Supplementary Fig. 2A). DVS_A and DVS_B have a good syntenic relationship with HSO except for several structural variations (SV) (Supplementary Fig. 2B). With DVS as the reference, a 99.9% mapping rate was achieved on the DVS whole-genome next-generation sequencing data. Higher mapping rates were achieved on 12 tested sweet orange datasets with DVS as the reference compared to HSO (Supplementary Fig. 2C and Supplementary Note 1). DVS_A and DVS_B have similar BUSCO completeness scores (98.4% and 98.3%) with HSO (98.4%), but HSO is on average 37.3 Mb larger. The extra regions in HSO are mainly located in its arbitrarily connected pseudochromosome chrUn (Supplementary Fig. 2B) which has an error rate of 0.21%. HSO has higher proportions of low-coverage and long tandem-repeat regions (Supplementary Fig. 2D) that were filtered and connected through repeat-unit resolving in DVS.

### Chromosomal origin and intra-genomic variations of DVS

We inferred the origins of the homologous chromosomal regions in DVS by comparing them to pummelos and mandarins (Supplementary Table 3). DVS_A contains ~290.3 Mb (97.1%) mandarin-origin (M) and ~ 8.7 Mb (2.9%) pummelo-origin (P) regions, and DVS_B has ~37.7 Mb (12.6%) M and ~ 261.9 Mb (87.4%) P regions (Fig. 1A and Supplementary Table 4). When combining the orthologous regions, there are approximately 254.8 Mb (84.7%) P/M, 37.5 Mb (12.5%) M/M, and 8.6 Mb (2.8%) P/P regions in DVS.

DVS_A and DVS_B share a 96.2% overall nucleotide similarity; 4 353 521 single-nucleotide variants (SNVs), 152 977 small indels (<50 bp), and 9989 SVs were detected between them (Fig. 1A). The SVs include 4923 insertions, 4563 deletions, 170 tandem duplications, 156 inversions, and 177 translocations. The seven largest SVs on chr1 and chr9, including two translocations and five inversions, are shown in Fig. 1B. The inter-chromosomal recombination between chr1A and chr9A is shared by all three Valencia sweet orange accessions sequenced in this study.

### DVS gene structure annotation and orthologous gene statistics

The DVS genome had approximately 49.0% (293.4 Mb) predicted as transposable elements (Supplementary Fig. 3). There were 55 745 protein-encoding genes annotated in DVS, including 27 807 on DVS_A and 27 938 on DVS_B. The DVS annotation has the highest (99.2%) BUSCO completeness among published citrus genomes, which is 6.2% higher than HSO (Supplementary Fig. 4A). The proteins from DVS and six other citrus assemblies were phylogenetically clustered into 24 817 ortholog groups. These groups were classified as high-quality (19328) and low-quality (5489) based on homology with plant proteins from other genera. DVS, DVS_A, and DVS_B have annotated genes from the most high-quality and the fewest low-quality ortholog groups (Supplementary Fig. 4B). We found 1386 high-quality groups with member(s) in DVS missing in HSO, and 549 are the other way around (Supplementary Table 5). Five hundred and thirty-three high-quality groups with members in both pummelo [15] and mandarin [4] genomes are missing either in DVS_A or DVS_B (Supplementary Fig. 4C and Supplementary Table 5).

We found 22 614 ortholog groups with members in both DVS_A and DVS_B, including 17 693 groups containing colinear allelic gene pairs (Supplementary Table 6). The bi-allelic genes from DVS_A and DVS_B share an average SNV density of 15.3 / kb in the exonic regions. A total of 12.7% (7069) and 15 721 (28.2%) genes have at least one allele affected by high-impact (disruptive such as frameshifting) intra-genomic small variants and SVs, respectively. DVS had 6933 (12.4%) hemizygous genes with only one allele either in DVS_A (3451) or DVS_B (3482) (Supplementary Table 7). These hemizygous genes are significantly overrepresented in biological processes including defense response, sexual reproduction, and hormone signaling (Supplementary Fig. 4D and Supplementary Table 8).

### Allele-aware RNA-seq pipeline and allelic expression patterns in sweet orange

We configured an allele-level RNA-seq analysis pipeline using DVS as the reference for sweet orange and other hybrids between pummelo and mandarin (Supplementary Fig. 5). With different reference genomes, the read mapping rates were variable for RNA-seq data from sweet orange, grapefruit, pummelo, and mandarin (Supplementary Table 9). For RNA-seq data of sweet orange, grapefruit, and mandarin, the highest overall and concordant mapping rates were achieved using DVS as the reference (Supplementary Fig. 6). With DVS as the reference, 73.1 ± 3.9% sweet orange RNA-seq reads were uniquely mapped (Supplementary Fig. 6). When applied to pummelo RNA-seq data, the overall and concordant mapping rates using DVS and DVS_B as the reference were only lower than the pummelo reference.

**Figure 2:**
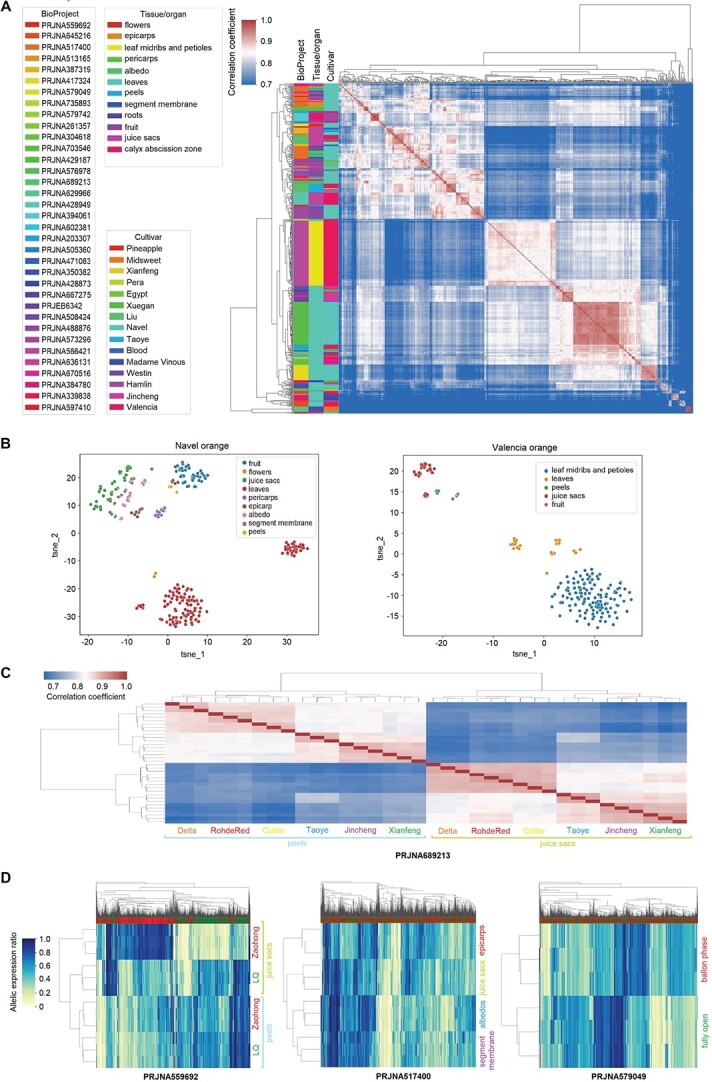
Allelic expression patterns (AEP) in sweet orange transcriptomes. (A) Hierarchical clustering based on AEP correlation coefficients (right) among 677 sweet orange transcriptomes. Each row or column represents a transcriptome. The AEPs are the allelic expression ratio profiling results for the transcriptomes, in which the expression ratios of each bi-allelic gene locus were quantified. Sixty-three transcriptomes were filtered from the 740 transcriptomes due to a low informative gene count. The colored bars on top of the heatmaps indicate alleles from DVS_A (red) and DVS_B (green). The NCBI BioProject IDs, cultivar types, and tissue/organ of the RNA-seq data are shown in the left. (B) T-SNE (t-distributed stochastic neighbor embedding) visualization of AEPs in different organs/tissues of Navel orange (left) and Valencia orange (right). tsne_1 and tsne_2 are the two features obtained through dimension reduction by T-SNE. In panels (C) and (D), transcriptomes of three biological replicates were analyzed under each condition. (C) Correlation heatmap and clustering of fruit peel and juice sac transcriptomes from six different sweet orange cultivars. Delta, Rohde Red, and Cutter belong to the Valencia cultivar group, and the other three are China local cultivars. (D) AEP heat map and hierarchical clustering of transcriptomes from three studies. Each row represents a transcriptome and each column represents a gene allele. Five hundred genes with the highest allelic expression ratio variances among the analyzed transcriptomes were used in making the graphs. Zaohong navel orange is a graft chimera of the LQ navel orange and *Citrus unshiu* Marc. In the middle panel, the transcriptomes were sampled at 220 days after flowering from Fengjie 72–1 navel orange. In the right panel, transcriptomes of Cara Cara navel orange flowers at two developmental stages were analyzed.

We carried out transcriptome profiling for 740 transcriptomes from 38 studies (NCBI BioProjects in Supplementary Table 10) to learn their allelic expression patterns (AEPs). We normalized the allelic expression quantity as the allelic expression ratios (proportions of the allelic reads in the corresponding gene reads) in AEP analysis (Supplementary File 1). A conservative estimation shows that 32.5% ± 0.08% of genes have a significantly biased allelic expression in sweet orange transcriptomes (Supplementary Fig. 7). We detected diversified AEPs in the transcriptomes that had significantly different allelic expression ratios on tens to hundreds of genes. Multiple AEP clusters related to source tissues/organs, cultivar types, different studies, and intra-study experimental conditions were identified through hierarchical clustering (Fig. 2A). Despite the complexity, the highest allelic expression correlations were observed among transcriptomes of the same tissues from the same cultivars (Fig. 2A). We then investigated the impact of different factors on the AEPs separately using the studies with single- or two- factorial designs.

### Impact of tissues and development stages on allelic expression

Different sweet orange organs/tissues generally alter the allelic expression ratios on hundreds of genes. In six sweet orange cultivars [16], the AEPs of juice sac and fruit peel transcriptomes were clustered into two large clades, with lower correlations between different tissue types than among the same tissues of different cultivars (Fig. 2C). Zaohong navel orange is a graft-chimera between navel orange and satsuma mandarin, with the L-1 histogenic layer being from satsuma, and L-2 and -3 from navel orange [17]. As expected, AEP analysis shows that Zaohong mainly expresses mandarin genes (DVS_A alleles) in the juice sacs (derived from L-1) but has similar AEPs with navel orange in the fruit peels (of L-2 origin) (Fig. 2D). With PRJNA517400 [18], we observed distinct AEPs among epicarp, albedo, segment membranes, and juice sacs of Fengjie 72–1 navel orange fruit at six different development stages (Fig. 2D). Epicarps had the most distinct AEPs at all six stages, while segment membrane and juice sac AEPs were the most similar.

**Figure 3:**
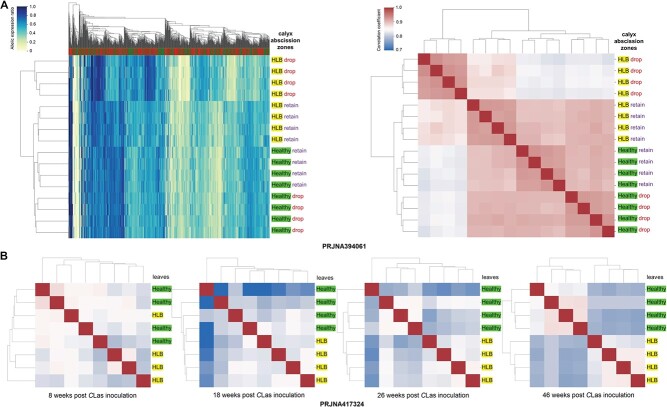
Citrus Huanglongbing- (HLB) induced allelic expression pattern alterations in sweet orange. Each row represents a transcriptome from the corresponding NCBI BioProject in the heat maps. (A) Hierarchical clustering based on allelic expression patterns (AEPs, left) and AEP correlation coefficients (right) among healthy and HLB-affected calyx abscission zones of Hamlin oranges. We used 1000 and 7500 genes with the highest allelic expression ratio variance in making the left and right panels, respectively. “Drop” and “retain” denote the abscission status of the fruit of the corresponding calyx abscission zones. (B) Correlation heatmap and clustering of leaf transcriptomes from healthy Washington navel orange seedlings and those affected by HLB.

Development is accompanied by an AEP shift in different sweet orange tissues. We observed significant allelic expression ratio alterations between balloon-stage and fully-open flowers of Cara Cara navel orange [19] (Fig. 2D). The AEPs of fruit transcriptomes from distinct development stages were clustered separately for Valencia sweet orange [20], Zaohong blood orange, and the 21^st^-century navel orange [21], respectively. We observed multi-step AEP transitions during Fengjie 72–1 navel orange (PRJNA517400) [18] fruit development in epicarp, albedo, segment membranes, and juice sacs (Supplementary Fig. 8). AEP transitions were also observed in juice sacs of Cara Cara navel orange [22] and peels of two navel oranges [23]. Analysis of PRJNA394061 [24] shows that fruit abscission induces AEP alterations in the calyx abscission zones (Fig. 3A).

### Allelic expression patterns in distinct sweet orange lineages

Different sweet orange cultivars (mutants) resulted in distinct AEPs in all analyzed datasets involving two or more cultivars (mutants) in this study (Supplementary Fig. 9), though the number of genes with significant allelic expression ratio alteration might vary. The juice sac or fruit peel AEPs from six sweet orange cultivars [16] were clustered in consistence with the cultivar types (Fig. 2C). The three Valencia cultivars (Delta, Rohde Red, and Cutter) shared higher AEP correlations among each other than with the three cultivars from China. Late Lane navel orange and its brown flavedo mutant Zong Cheng had distinct AEPs in fruit peels at five different fruit development stages. We observed 36.2 ± 9.5% allelic expression ratio decreases of 19 neighboring genes on chr3A in Zong Cheng and found a deletion in its genome sequencing data, which was not reported in the original study [23]. Three acidless oranges [5] and two navel oranges [25] were distinguished via fruit and juice sac AEPs, respectively.

AEP alterations were observed in most genetically engineered plants compared to the wild type. All six analyzed sweet orange transgenic lineages [26–28] with gene overexpression had different AEPs from the wild type (Supplementary Fig. 10). The observed AEP alterations could either be related to the manipulated genes or somatic mutations randomly induced by genetic engineering. Two sweet orange lineages overexpressing *CsGH3.1* and *CsGH3.1 L* [26] had distinct AEPs. The AEPs of sweet orange lineages OE-5 and OE-6 both overexpressing *CsWRKY22* [28] were not significantly different, while the AEPs of OE-2 and OE-15 overexpressing *CsLOB1* (PRJNA670516) [27] were distinct by clustering. We observed allelic expression ratio increases of 140 neighboring genes on chr7A by 48.4 ± 13.7% only in OE-15 (Supplementary Fig. 10) resembling AEP alterations induced by deletions detected in genomes of Zongcheng and T78 below. The AEPs of two sweet orange lineages (RI-D3 and RI-D4) with RNA interference of *CsLOB1* [27] were not distinguished from the wild-type transcriptomes.

### Impact of citrus Huanglongbing on sweet orange allelic expression patterns

HLB causes symptoms on both citrus leaves and fruit in the field [7], and we found AEP alterations in different sweet orange tissues associated with HLB infection. Analysis of PRJNA394061 [24] showed that healthy calyx abscission zones with HLB had altered AEPs compared to the healthy tissues (Fig. 3A). In HLB-affected calyx abscission zones with fruit retained and dropped, we found 2612 and 3739 genes with significant allelic expression ratio alterations compared to the corresponding healthy tissues, both enriched in defense response, lipid metabolic process, and organic acid catabolic process (Supplementary Table 11).

We have not observed AEP alterations related to HLB in leaves at 1 and 5 days post-inoculation (PRJNA645216) [29]. In PRJNA417324 [30], the leaf AEPs of trees inoculated with *C*Las gradually diverged from the healthy controls from 8 WPI (weeks post-inoculation) to 46 WPI. The AEPs in the inoculated trees start differing at 8 WPI when lower AEP correlations have been observed among the transcriptomes with HLB, implying a putative unstable stage of the disease with diversified impacts on different branches. At 26 WPI, the HLB AEPs are already clustered together in a clade. At 46 WPI, 1008 genes with significant allelic expression ratio alterations (FDR < 0.10) are enriched in defense response, lipid metabolic process, sterol biosynthetic process, and carbohydrate catabolic process (Supplementary Table 12). These results indicate the impact of HLB on sweet orange AEP is a long-term process.

### Irradiation-induced Valencia sweet orange mutants

**Figure 4:**
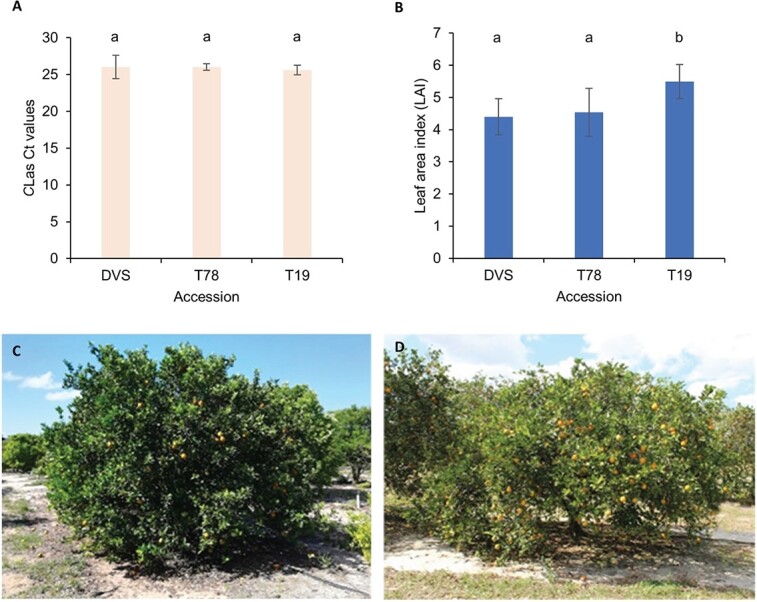
*C*Las titer and citrus Huanglongbing (HLB) symptoms on investigated Valencia sweet orange accessions. (A) *C*Las titers of the DVS, T78, and T19 trees. The titer of *C*Las is represented by the Ct. values of qPCR tests using the 16S rRNA primers. (B) Leaf area indexes of the investigated trees. The *C*Las titers and leaf area indexes were tested on 5 to 6 different sections of each tree for 2 DVS, 2 T78, and 4 T19 trees (including SF). One largest and one smallest values of each tree were removed before calculating the mean Ct. and leaf area index values and the standard deviations (error bars in panels A and B). The lower-case letters a and b on top of the bars denote statistically different (p < 0.05) groups. (C) and (D) Photos of an HLB-affected T19 tree and an HLB-affected DVS tree taken in April 2019, respectively. Note the relative differences in tree size, canopy density, and color of the foliage.

We took advantage of the allelic information of the DVS assembly to probe the possible underlying molecular mechanisms in an HLB-tolerant sweet orange mutant. Most trees growing in the same field trial location as the mutants were killed by HLB or removed because of severe decline caused by HLB. Only six trees from two original selections, four from T19 and two from T78, are still growing in the grove. The four T19 trees included one with a lost tag (SF), which was proven identical to the other three T19 trees by whole-genome sequencing, as described in the following section. Though similar *C*las titers [31] were detected on T19, T78, and DVS (Fig. 4A) indicating equivalent infections, the T19 trees had significantly greater (p < 0.01) leaf area indexes than DVS and T78 (Fig. 4B). The four T19 trees are still healthy (Fig. 4C) and have less severe symptoms than DVS (Fig. 4D) and the two T78 trees.

### Somatic structural mutations in the sweet orange accessions

We obtained over 280× Pacbio continuous long reads for each of T19, SF, and T78. We have not detected any SVs between T19 and SF, and three somatic SVs (TRA7, TRA44, and INS16) are shared by them (Supplementary Table 13), proving that SF was derived from the original T19 branch. TRA44 and TRA7 are both complex translocations involving inversion and inter-chromosomal recombination (Supplementary Fig. 11). The genic regions of four genes and the promoter region of another gene have been truncated by TRA7 and TRA44 (Supplementary Table 14). INS16 is a 5086 bp Mutator–like transposable element (MULE) insertion in chr2A. Four lineage-specific mutations were identified in T78 (Supplementary Table 13 and Supplementary Fig. 11), including TRA22, a chromosomal recombination event between chr5B and chr1B; INV17, an 88 911 bp inversion on chr2B that truncated two genes; DEL58, a 1.8 Mb deletion on chr8B that has deleted 138 genes and truncated 1 gene; DUP52, an 5617 bp tandem duplication on chr8A. TRA7, INV17, TRA22, TRA44, INS16, and DEL58 were verified through PCR amplification (Supplementary Fig. 12). We also detected forty SVs between DVS and both T19 and T78 that were inferred to be spontaneous mutations (Supplementary Table 15 and Supplementary Note 2).

### Allelic expression alterations in the sweet orange mutants

**Figure 5:**
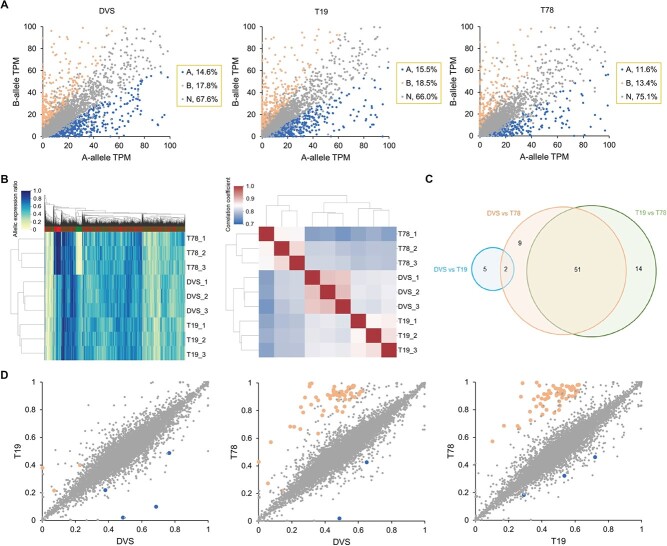
Biased allelic expression and allelic expression ratio alteration in DVS, T19, and T78. (A) Allelic expression in DVS, T19, and T78 transcriptomes. Each dot represents a DVS bi-allelic gene locus. A-allele and B-allele denote the corresponding alleles of a gene in DVS_A and DVS_B. The allelic expression quantity was normalized as transcripts per million (TPM). Genes with significantly biased allelic expression are colored blue (mainly expressing A-allele) and light red (mainly expressing B-allele), respectively. (B) Hierarchical clustering of DVS, T19, and T78 based on their allelic expression patterns. The transcriptomes in the left panel were clustered using one thousand genes with the highest allelic expression ratio variance. The scale bars denote the corresponding colors of the allelic expression ratios in the left panel and Pearson correlation coefficient r in the right panel. (C) The distribution of genes with significant allelic expression ratio alterations among DVS, T19, and T78. (D) Scatter plots showing the corresponding A-allele expression ratios of genes among DVS, T19, and T78. Each dot represents a DVS gene. The axes denote the A-allele expression ratios in the corresponding accessions, with 0 indicating 0% A-allele expression and 1 indicating 100% A-allele expression. Genes with significant A-allele expression ratio increase and decrease in the horizontal axis accession compared to the vertical axis accession are colored blue and light red, respectively.

We carried out transcriptomic profiling for DVS, T19, and T78. In T19, 1726 alleles were significantly upregulated, and 1503 were downregulated compared to DVS and T78 (Supplementary Table 16). In T78, 1054 upregulated and 1161 downregulated alleles were detected compared to DVS and T19 (Supplementary Table 17). For most alleles with significantly altered expression in T19 (67.4%) or T78 (73.8%), the expression of their alternate alleles was not significantly different. Significantly biased allelic expression (≥1.5-fold allelic difference) is observed in 32.4%, 34,0%, and 25.0% of the 10 737 tested genes in DVS, T19, and T78 transcriptomes (Fig. 5A), including 1962 in common among them (Supplementary Table 18).

**Figure 6:**
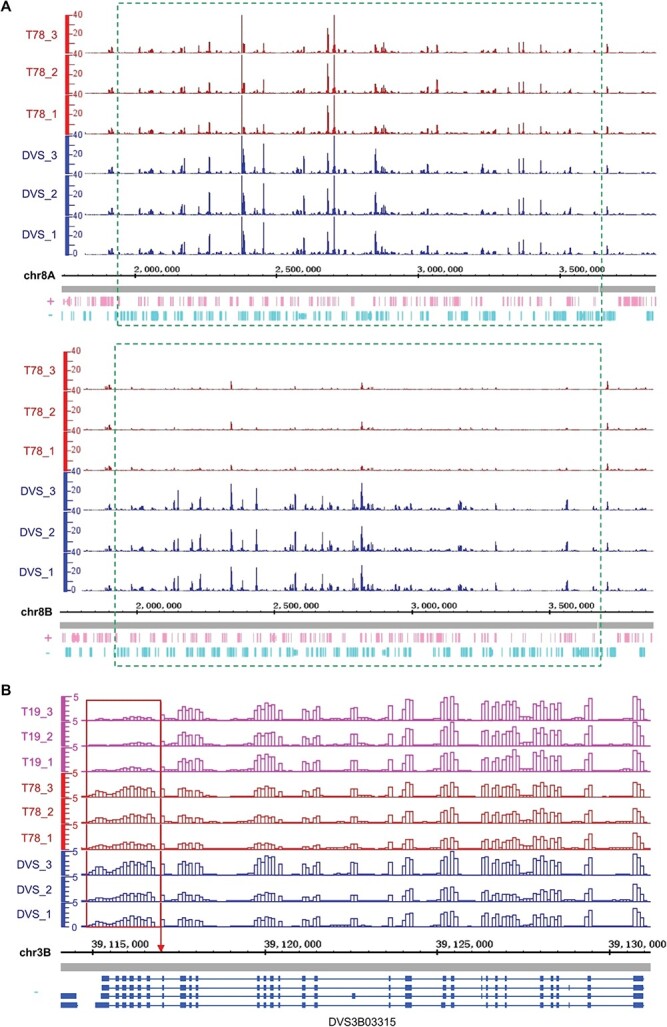
Allelic expression alterations directly induced by structural mutations. In both (A) and (B), the genomic regions were split into 100 bp continuous non-overlapping windows. The vertical axes show the normalized uniquely mapped RNA-seq read counts in each window using the counts per million reads mapped (CPM) method. The horizontal axes denote the coordinates (bp) in the DVS genome. (A) Allelic expression in the deleted region of DEL58 (bottom panel) and its allelic region (top panel) in DVS and T78. DEL58 is a 1.8 Mb deletion on chr8B only detected in T78. The green dashed frames denote the deleted region (bottom panel) and its allelic region on chr8A (top panel). (B) Regional expression alteration of DVS_B *CsXPO1* (DVS3B03315) truncated by TRA7. Different transcript isoforms are shown at the bottom, with blue rectangles representing exons and lines indicating introns. The red frame marks the truncated 3′ region of *CsXPO1* in T19, and the red arrow points to the chr3B:39117014 break endpoint of TRA7.

DVS, T78, and T19 transcriptomes had distinct AEPs (Fig. 5B). We detected significant allelic expression ratio alterations (≥1.5-fold) among DVS, T19, and T78 on eighty-one genes (Fig. 5C, D and Supplementary Table 19). Sixty-two of these genes were located in the 1.8 Mb deleted region of DEL58 (Supplementary Table 19). The expression of the deleted alleles by DEL58 was almost eliminated (Fig. 6A), while their alternate alleles were mostly (115/118) not significantly affected. The allelic expression ratio of DVS_A *CsOPT9* interrupted by a 25 857 bp insertion (INS34 in Supplementary Table 19) in DVS has been reduced to 0.1%, compared to the 38.1% in T19 and 43.0% in T78 (Supplementary Table 19). A few alleles directly affected by the somatic SVs had significantly different expressions in the mutants, though no significant allelic expression ratio alteration was detected (Supplementary Table 14). In T19, the expression of *DVS7B01006* (encoding a GPI-anchored adhesin-like protein) that was truncated by TRA7 was significantly downregulated. The 3′ end of DVS_B *CsXPO1* trimmed by TRA7 had significantly lower expression compared to DVS and T78, while the 5′ region was not significantly altered (Fig. 6B).

### The HLB-tolerance mechanism in T19

**Figure 7:**
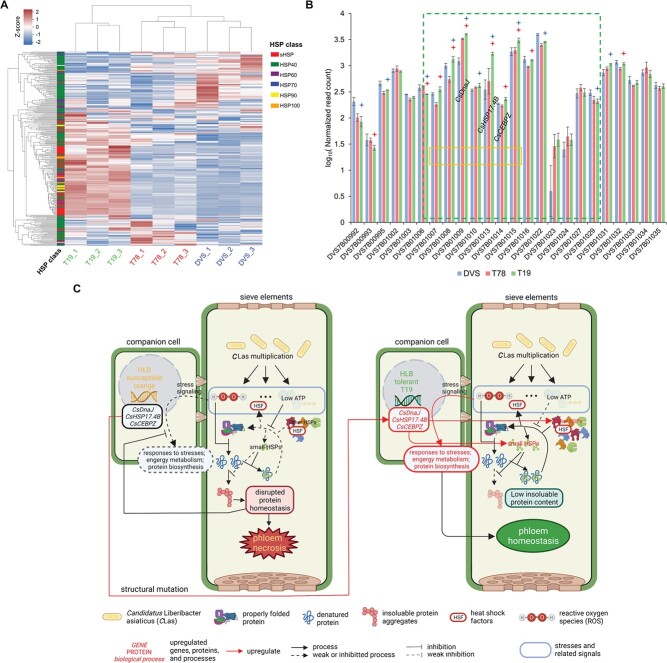
Transcriptomic alterations and molecular mechanisms related to the high citrus Huanglongbing (HLB) tolerance of T19. (A) Heat map showing RNA-seq expression quantification of heat shock protein genes (*HSP*s). DESeq2 normalized RNA-seq read counts were utilized in making panels A and B. Each column shows the z-scores of normalized read counts of an *HSP* in the nine transcriptomes. The hierarchical clustering dendrograms were produced based on the Euclidean distances (Z-scores) among the transcriptomes (right) and the HSP genes (top). (B) Histogram showing the normalized gene expression across the translocated segment (green dashed-frame) by TRA7. Only genes with an average read count of ≥3 per transcriptome are shown. Blue and red stars denote being significantly (FDR < 0.1) different in T19 from DVS and T78, respectively. The orange frames indicate the seven genes with upregulated expression (*p* < 0.05) in T19 compared to both DVS and T78. (C) Hypothesized molecular mechanisms for HSP-related HLB tolerance of T19. The multiplication of *C*Las inside citrus phloem cells causes the burst of multiple stresses. In the susceptible sweet orange (left), the basal HSP levels are too low to deal with the stress-induced denatured proteins, resulting in the protein homeostasis collapse which paralyzes phloem metabolism and disables stress-induced defense responses. In the tolerant mutant T19, the expression of *CsDnaJ* (DVS7B01009 in panel B), *CsHSP17.4B* (DVS7B01013), and *CsCEBPZ* (DVS7B01014) are upregulated by the structural mutation TRA7, which increases basal HSP level and keeps phloem protein homeostasis under the stress burst, allowing the induction of the stronger stress-induced responses observed in T19 transcriptomes.

T19 had significantly enriched gene upregulation in responses to multiple stresses (including heat, osmotic, oxidative, and nutrition-level), energy metabolism (including mitochondrial metabolism and photosynthesis), ribosome biogenesis, and translation activities compared to DVS and T78 (Supplementary Table 20). Moreover, hairpin precursors (pre-) of three stresses related microRNAs were differentially expressed in T19; pre-csi-MIR160b,c [32] were significantly upregulated, pre-csi-MIR398b [33] and pre-csi-MIR396c [34] were significantly downregulated in T19 (Supplementary Table 21). We did not observe upregulation of immunity responses or responses to biotic stresses in T19. Ninety-two of all 274 expressed *HSP*s were significantly upregulated in T19 compared to DVS and T78 (Fig. 7A and Supplementary Table 22). *HSP90s*, *HSP70s*, *HSP100s*, and small *HSPs* (*sHSP*s) were enriched over 10-fold (FDR < 0.05) among the upregulated genes (Supplementary Table 23). Among them, s*HSP* had the largest number (44) of upregulated genes and also the highest mean upregulation level (10.6-fold on average). Moreover, ten enzyme-encoding genes involved in reducing reactive oxidative species (ROS) were significantly upregulated in T19, including 7 *CsAPX* family members, *CsCSD1*, and 2 *CsP2* (Supplementary Table 16). Considering reducing ROS alone could relieve HLB symptoms [35], the widespread enhanced stress responses only observed in T19 should explain its high HLB tolerance (Supplementary Fig. 13).

Somatic mutations are assumed to be the ultimate cause of the transcriptomic difference, thus we searched for the stress response-related genes affected by the SVs. One terminal of the segment translocated by TRA7 is significantly (FDR = 0.015) enriched in upregulated alleles (Fig. 7B). We found three HSP-related genes in this region all upregulated in T19, including an s*HSP* (*sHSP17.4B*), an *HSP40* (*CsDnaJ*), and a CCAAT enhancer-binding protein-encoding gene (*CsCEBPZ*) (Fig. 7B, C). HSPs are known to counter ROS and play important roles in plant stress responses [36, 37]. Considering the susceptible DVS and T78 could not bring such stress responses, we hypothesize that the upregulation of the HSP-related genes enables the stress responses in T19 by preventing the phloem protein homeostasis from collapsing under stress burst from *C*Las multiplication (Fig. 7C).

## Discussion

We assembled a phased chromosome-level sweet orange genome with improved completeness, accuracy, and gene structure annotation compared to the recently updated HSO genome [5]. We have found a high level of intra-genomic variance in DVS, with more than four-fold SNVs compared to previously reported using the next-generation sequencing method [1]. The abundant hemizygous genes and high allelic variance level in DVS indicate the genetic redundancy of sweet orange as a diploid has been discounted considerably. DVS enables allele-level mutation identification and gene expression quantification in sweet orange, and will also facilitate allele-specific protein expression analysis [38] and allele-specific genetic engineering [39]. Many citrus cultivars contain genetic materials from both pummelo and mandarin [2, 3], making DVS a very important tool for genetic analyses within *Citrus*.

As the lengths of sequencing reads improved [40], the high intra-genomic heterozygosity, such as found in sweet orange, could be utilized as an advantage in phased genome assembly. We adapted the assembly parameters to the allelic difference level of sweet orange and assembled most genomic regions directly into two haploid contigs. Our method avoided mapping-based phasing in the highly heterozygous genomic regions and has achieved a very low hamming error rate. Genetic maps [1, 2, 41] or the Hi-C technology [5, 42] have been utilized in the scaffolding step of the citrus genome assembly. This study shows that for genomes with diverged repeat units among long-tandem repeat regions, the long sequencing reads could have contained enough information for chromosome-level scaffolding. CANU’s capacity to resolve high-similarity repeats [43] is important for the successful application of our method.

The phased DVS assembly allows precisely resolving the genetic composition and origin of sweet orange. Two whole-genome sequencing-based studies had different inferences on the genomic composition and origin of sweet orange [1, 2]. Xu et al. (2013) estimated the di-haploid sweet orange genome with ~1/4 pummelo and ~ 3/4 mandarin genomic contributions and inferred it to have originated as (P × M) × M. The genomic composition of the diploid sweet orange identified in this study is close to that found by Wu et al. (2014). The chloroplast and mitochondrial DNA indicated that sweet orange has the pummelo cytoplasm [44]. Accordingly, sweet orange most likely had either a [(P × M) × P] × P or a (P × M) × P as its maternal parent, and a mandarin which contained a small proportion of pummelo introgression (containing ~2.8% pummelo nuclear DNA) should be its paternal parent.

This study has revealed a large number of AEPs but is still far from the full scenery in sweet orange. Previously, the knowledge of AEP diversity in sweet orange was mostly non-existent, since allelic expression has rarely been studied [45, 46] due to the lack of a high-quality phased reference genome. The diversified AEPs in different organs/tissues at different development stages possibly explain why sweet orange has many specific characteristics different from its parental species. The wide existence of allelic expression ratio alterations among sweet orange cultivars (mutants) is not only related to somatic mutations. They also imply a high abundance of cis variations [47] in the sweet orange genome. Due to the abundant cis variations, novel somatic mutations might induce a butterfly effect on AEP, which partially explains why sweet orange and other citrus inter-specific hybrids have a high frequency of bud sport selections.

Most irradiation-induced and spontaneous somatic SVs have developed through different molecular mechanisms. Irradiation could cause a wide variety of DNA lesions, among which double-strand breaks are the most relevant to structural mutations [48, 49]. In this study, five of the seven putative irradiation-induced SVs in T19 and T78 involved double-strand break repair in the non-homologous end-joining manner [50]. TE activity has been reported to be the primary source of spontaneous SVs in a few plants [51, 52]. A few bud sports from sweet orange and other citrus fruit types have been distinguished by TE polymorphism [53–55]. In 114 sweet orange accessions, TE insertions accounted for 40.1% of the large somatic insertions detected [5]. This study shows that the spontaneous SVs detected are mainly (31/40) derived from the insertion of three MULEs (Supplementary Table 15), indicating they might be hyperactive and have played an essential role in the formation of some sweet orange cultivars.

SVs have been reported to cause differential gene expressions in a few species [56, 57]. The retrotransposon insertion in the promoter region caused the *Ruby* gene to be expressed under cold stress in blood sweet orange [6]. Diversified effects of SVs on gene expression have been observed in this study. The expression of deleted alleles in T78 was generally eliminated as expected, and their alternate alleles’ expression was mostly unaffected. The case for a truncated gene was more complicated, which could either be entirely downregulated or only have a 3′ end downregulation. Chromosomal rearrangements could affect the expression of adjacent genes by causing changes in chromatin topology, but their effects remain difficult to predict [58]. We found most chromosomal rearrangements in T19 and T78 had no noticeable impact on the expression of adjacent genes except for TRA7.

Multiple mechanisms have been associated with HLB resistance/tolerance in citrus (Supplementary Fig. 13) [10, 11, 14, 35, 59–63]. The primary cause of HLB symptoms is the dysfunctional phloem induced by *C*Las infection [14, 64]. ROS accumulation has been reported to play an important role in HLB symptom development, and Ma et al. (2022) inferred it to be caused by chronic immune responses in the phloem tissue [35]. However, there is no clear proof that *C*Las could be recognized by citrus within the phloem, and we did not observe different immunity responses between T19 and the susceptible accessions (DVS/T78). Moreover, the chronic accumulation of ROS cannot explain the different stress responses between T19 and DVS/T78. Considering plant sieve elements are cells specialized for transporting sugars throughout the plant which lack nuclei and have very few organelles, the metabolic wastes and ATP/nutrition consumptions from *C*Las multiplication should be able to cause a burst of stresses in the phloem (Fig. 7C). A reasonable explanation for the different stress responses between T19 and DVS/T78 is that the stress burst paralyzes the phloem protein system of DVS/T78 but is endured by the T19 due to the upregulation of HSP-related genes induced by TRA7. The immunity responses including callose deposition and programmed cell death induced by *C*Las microbe-associated molecular patterns [65] are inferred to occur after the stress-induced phloem necrosis in the susceptible citrus (Supplementary Fig. 13). HSPs are molecular chaperones responsible for keeping cellular protein homeostasis under abiotic and biotic stresses [36, 37]. The three upregulated HSP-related genes putatively have enhanced the basal tolerance of denatured proteins in the T19 phloem. *CsHSP17.4B* encodes an sHSP that binds denatured proteins and prevents protein aggregation in an ATP-independent manner [66]. *CsDnaJ* (*HSP40*) stimulates the ATPase activity of HSP70 heat-shock proteins [67], and its upregulation has potentially reduced the impact of lowered ATP levels due to *CLas* consumption. *CsCEBPZ* encodes a CCAAT enhancer-binding protein, and CCAAT boxes are found in the promoters of many plant HSPs [68, 69]. Our study brings a new hypothesis on HLB symptom development and indicates the basal HSP (particularly the ATP-independent sHSP) level might have played a key role in the HLB tolerance of citrus.

## Methods

### Plant materials

Valencia sweet orange buds were exposed to 50 Gy gamma irradiation, and more than 1200 trees were produced by budding onto Volkamer lemon rootstock seedlings and planted in the field in 1992, as part of a mutation breeding project. From these trees, 6 were identified as bearing nearly seedless fruit including T19, SF, and T78. These were subsequently repropagated onto Carrizo citrange rootstock and at least 2 of T78 and 3 of T19 were planted in the field near Lake Alfred, FL in the summer of 2000. They along with DVS have grown in the field under the same management conditions since then. DVS and OVS are different Valencia orange trees, the former being the tree used to produce the genome assembly reported here, and the latter being the budwood source to produce the irradiated Valencia population from which T19, T78, and SF were selected. The SF tree was known to be grafted from one of the original nearly seedless selections from the same experiment, but the identification tag was lost, so its clonal identity was uncertain. HLB was first detected in this field location in 2008, and by 2010 virtually all trees were showing symptoms of disease. These specific individuals, although also exhibiting HLB symptoms, were first noted for their obvious superior performance and substantially better appearance compared with all other nearby trees of standard Valencia that were in severe decline, as well as a wide range of other materials from the breeding program likewise in severe decline, in 2017. T19 trees have retained their tolerant phenotype, but T78 trees have gradually declined since first noted.

### Measurement of leaf area index and *C*Las titer

The leaf area index (LAI) was measured using AccuPAR LP-80 (Meter Group, Pullman, WA, USA) near solar noon in June 2021. The external photosynthetic active irradiation (PAR) sensor was placed in a nearby open area, and the LP-80 instrument PAR probe was placed under the canopy of each tested tree. The LP-80 computed LAI from the PAR readings and χ (leaf angle distribution parameter). The default χ parameter (χ = 1) was applied. On average, 6–7 measurements per tree were taken around each tree.

For *C*Las titer measurement, DNA was extracted from leaf midribs and petioles of each tree using the Plant DNeasy Mini Kit (Qiagen, Valencia, CA, USA) according to the manufacturer’s instructions. qPCR quantification of the *C*Las titer using 16S rRNA primers was carried out as described by Li et al. (2006). qPCR was performed on an Agilent Mx3005P (Agilent Technology Inc, Waldbronn, Germany) real-time PCR system with the Brilliant III Ultra-Fast QPCR Master Mix (Agilent Technology Inc, Waldbronn, Germany).

### DNA and RNA extraction

For next-generation sequencing (NGS) and PacBio sequencing, young leaves of DVS, T78, T19, and SF (PacBio sequencing only) were collected from new flushes in April 2018 and April 2019, respectively. We used the CTAB method [70] to extract genomic DNA for NGS. For PacBio sequencing, genomic DNA was isolated using Nanobind Plant Nuclei Big DNA Kit (Circulomics Inc., Baltimore, MD, USA) following the manufacturer’s instructions.

For RNA extraction, mature leaves were collected from three different tree parts for DVS, T19, and T78 as replicates. In total, nine RNA samples were extracted using TRIzol™ and RNA Purification Kit following the manufacturer’s protocol (ThermoFisher, Waltham, MA, USA). RNA was further purified using the TURBO DNA-free™ kit (ThermoFisher, Waltham, MA, USA) to eliminate genomic DNA. Both NanoDrop Spectrophotometer (NanoDrop Technologies, Wilmington, DE, USA) and Agilent 2100 Bioanalyzer (Agilent Technologies, Waldbronn, Germany) were used to assess the RNA quality and quantity.

### De novo assembly of DVS and the mutants

Whole-genome PacBio continuous long reads (CLR) were obtained for DVS, SF, T78, and T19 on the PacBio Sequel II system (Pacific Biosciences, Menlo Park, USA). One-hundred-fold coverage of corrected reads (N50 = 42.0 kb) was used. A minimum of 98.5% overlap identity was required in the assembly step to reduce collapsed assembly in heterozygous regions. We carried out de novo assembly of DVS using MECAT2 [71] with four different minimum read overlap lengths (500 bp, 2 kb, 5 kb, and 10 kb). A 2 kb minimum read overlap length was identified as optimal since the assembly achieved the second-largest N50 and the largest assembly size. The assemblies’ accumulative length and contig N50 were assessed using QUAST v5.1 [72]. De novo assembly of the three mutants was carried out by MECAT2 using the optimal parameters observed for DVS (Supplementary Table 24).

We also carried out de novo assembly of DVS using CANU v2.1 [43] with the optimal settings (correctedErrorRate = 0.015, minOverlapLength = 2000, genomeSize = 340 m, and corOutCoverage = 100). CANU produced ~26.6 Mb more sequences mainly derived from repetitive regions. The contigs in the CANU assembly were connected into pseudochromosomes through (1) phased assembly of the collapsed and expanded regions; (2) resolving the repeat units in long tandem repeat regions (Supplementary Fig. 1). In the process, each contig in the CANU assembly was mapped against the remaining contigs using minimap2 v2.17 [73] with option -x asm20 to detect orthologous regions. The sequencing depth across the assembly was output using BEDTools v2.29.2 [74] in 1 kb windows. The unphased regions and putatively collapsed regions were subjected to phased assembly using pb-falcon v2.24 [75] with our bash script and configuration files (accessible from https://github.com/TheLuoFengLab/DVS-assembly-and-allele-aware-RNAseq-pipeline.git). We used the BLASTN algorithm in NCBI blast+ v2.5.0 [76] to align contig terminal segments for repeat unit identification. A circular mitochondrion genome and a circular plastid genome were manually recovered by aligning and connecting several contigs with high coverage. The chromosomes were named in concordance with the haploid CCL genome [2]. Using the CLR reads, three rounds of polishing were carried out by pbmm2 and arrow in GenomicConsensus v2.3.3 (Pacific Biosciences, Menlo Park, USA). The phylogenetic origin of the chromosomal regions and switch errors were inferred using twenty mandarin and twenty pummelo whole-genome NGS data sets (Supplementary Table 3) downloaded from the NCBI database using the method described in Supplementary Note 3.

### Hamming error rate estimation

The paternal and maternal parents of sweet orange are unknown, so we estimated the hamming error rate across the genome based on its di-haploid offspring HSO [5]; 100 × sequencing reads were simulated without sequencing error based on the HSO genome by wgsim (acquired on 06/15/2021 from https://github.com/lh3/wgsim). The simulated reads were mapped to DVS using minimap2 v2.17. The simulated reads uniquely mapped to DVS_A (*C_A_*) and DVS_B (*C_B_*) were counted in 20 kb continuous windows with 15 kb overlap across the HSO genome. The hamming error rate was calculated as the minimum of *C_A_* and *C_B_* divided by the sum of *C_A_* and *C_B_* in each window. HSO is from a di-haploid offspring of sweet orange, thus the windows overlapped with putative chromosomal recombination loci in HSO, identified with surrounding windows switching from *C_A_ > C*_B_ to *C_B_ > C_A_* or vice versa, were excluded from the calculation.

### Quality evaluation and comparison between DVS and HSO

HSO and DVS were aligned using minimap2 v2.17. The dot-plot was then drawn by D-GENIES v1.2.0 [77]. For assembly quality assessment, we obtained 26–30 × pair-end sequencing reads (150 bp × 2) from Illumina HiSeq 2500 (Illumina, San Diego, CA, USA) for DVS, T19, and T78. The K-mer-based completeness and the error rates of the assemblies were assessed by Merqury v1.3 [78]. The core gene completeness test was carried out by BUSCO v5.0.0 [79] using its eudicots_odb10.2019-11-20 database.

For the mapping rate test, we downloaded twelve whole-genome NGS data sets of different sweet orange cultivars (Supplementary Table 25) from NCBI. They were aligned to DVS and HSO by BWA v0.7.17 with the default parameters. Then we obtained the mapping rates using SAMtools v1.10.

To analyze the sequencing depth homogeneity across the HSO assembly, we downloaded its whole-genome PacBio continuous long reads (SRR5838837) [5] from NCBI. The long reads of DVS and HSO were mapped to them respectively by minimap2 v2.17. The sequencing depths across the two assemblies were output using BEDTools v.2.29.2.

### Intra-genomic variation detection and annotation

We aligned DVS_B to DVS_A using minimap2 v2.17 and called the small variants using BCFtools v1.10 with the consensus model [80]. Only regions with one-to-one unique alignments were used to call the variations. SNVs or indels within 10 bp distances of other indels were filtered. For SV detection, we carried out whole-genome alignment between DVS_A and DVS_B using Mummer v4.0.0 [81], and the SVs were called by MUM&CO v2.4.2 [81]. The circular graph (Fig. 1A) showing the distribution of the variations was drawn using Circos v0.69–9 [82]. Genetic variant annotation and functional effect prediction for the small variants were predicted by SnpEff v5.0 [83], which defined the categories of high, moderate, low, and modifier impact variants. Genes overlapping the SVs were analyzed with BEDTools v2.29.2.

### Annotation of the DVS genome

TEs were predicted in the DVS genome using both RepeatModeler v2.0.1 [84] / RepeatMasker v4.1.1 [85] and the Extensive de-novo TE Annotator (EDTA) v1.8.3 pipeline [86]. Unclassified TEs were subjected to convolutional neural networks-based classification by DeepTE using the plant model [87].

The DVS genome was soft-masked using RepeatMasker v4.1.1 and the non-redundant TE library from the EDTA pipeline. *Ab initio* gene prediction and transcriptome data assembly-based methods were applied in combination to annotate gene models in the genome. Eighty sweet orange transcriptomic RNA-seq data sets (Supplementary Table 26) from multiple tissue types were downloaded from NCBI and mapped to DVS using HISAT2 v2.2.1 [88] for RNA-seq evidence. The UniProtKB/Swiss-Prot plant database (accessed on 10/13/2020) [89] was used to generate protein hints with GenomeThreader v1.7.3 [90]. Then GeneMark-EP+ v4.65 [91] and Augustus v3.4.0 [92] were trained based on the RNA-seq and protein hints using BRAKER v2.1.5 [93]. For transcriptome assembly-based annotation, RNA-seq reads uniquely mapped to DVS_A or DVS_B were separated into two sets, with reads equally mapped to DVS_A and DVS_B added to both sets. RNA-seq read assembly and transcript screening were then carried out for DVS_A and DVS_B respectively with Mikado v2.0 [94].

### Annotation completeness comparison among citrus assemblies

To compare the completeness of gene structure annotations, the protein sequences encoded by the first transcript of all genes were output for DVS, DVS_A, DVS_B, HSO [5], Clementine (*Citrus clementina* Hort. ex Tan.) [2], box orange [*Atlantia buxifolia* (Poir.) Oliv.], Ichang papeda (*Citrus ichangensis* Swingle), citron (*Citrus medica* L.), pummelo [15], mandarin [4], kumquat (*Citrus hindisii* Champ. ex Benth.) [95], ZK (*Citrus trifoliata* L.) [41], and PTR (*C. trifoliata*) [42]. The protein sequences were tested against the eudicot core gene set (eudicots_odb10.2019-11-20) by BUSCO v5.0.0 [79].

### RNA-seq mapping rate tests

The RNA-seq data mapping rate was compared with different citrus assemblies as the reference. A masked DVS version was generated by masking the allelic genes with <3 SNVs / kb in the exonic regions except for one allele in DVS_A. The separately tested DVS_A and DVS_B were not masked. Forty RNA-seq data sets from sweet orange, grapefruit, mandarin, and pummelo were downloaded from NCBI (Supplementary Table 9). The RNA-seq data were mapped to the assemblies by HISAT2 v2.2.1, which reported the overall, concordant, and unique mapping rates.

### Analysis of orthologous gene groups in citrus

The protein sequences from the DVS_A, DVS_B, HSO, Clementine, pummelo, mandarin, kumquat, and trifoliate orange (PTR) genomes were phylogenetically clustered into ortholog groups using OrthoFinder v2.5.2 [96]. The first protein in each ortholog group was searched against the PANTHER v16 [97] database using MMseqs2 v12-113e3 [98]. High-quality ortholog groups were identified by requiring two criteria: (1) including members from at least three citrus genomes (DVS_A and DVS_B only counted once); (2) having at least one target hit in the PANTHER database with the alignment covering ≥30% of both the query and the target sequences and an E-value <1.0E-3. Those groups that did not fully meet the criteria were regarded as low-quality. Co-linear orthologous (allelic) genes on DVS_A and DVS_B were inferred using MCScanX [99].

### Detection of somatic SVs in the radiation-induced mutants

Assembly and mapping-based strategies were combined in detecting somatic structural variants. The assemblies of the mutants were aligned to DVS, and candidate SVs were called by MUMMER v4.0.0. We obtained a set of false-positive SVs by aligning the four MECAT assemblies of DVS. In the mapping-based method, the PacBio continuous long reads were mapped using Minimap v2.17, and the SVs were called using Sniffles v1.0.12 [100] with a requirement of at least ten zero-mode waveguides support. Then we compared the SVs from the two strategies. Maximum margin distances of 50 bp for breakpoint ends of translocations, and 50 bp or 10% of the SV lengths (whichever smaller) for deletions, insertions, tandem duplications, and inversions, were allowed for the SVs from the two strategies to be considered the same. SVs detected by both methods were subjected to further analysis. We designed primers for 6 SVs and carried out ordinary PCR amplification for verification (Supplementary Table 27).

### RNA-seq, miRNA-seq, and differential expression analysis

Whole-transcriptome sequencing and microRNA sequencing were carried out by BGI Genomics (Shenzhen, China). The sequencing via Illumina HiSeq 4000 (Illumina, San Diego, CA, USA) produced >5 Gb clean pair-end (150 × 2) reads for each sample.

The masked DVS in RNA-seq mapping rate tests was used as the reference in RNA-seq analysis. Salmon v1.4.0 [101] was applied in read mapping and counting, with multi-mapped reads assigned by the expectation–maximization algorithm. Read count normalization and differential expression tests were carried out by DESeq2 v1.30.1 [102]. Genes with significantly (FDR < 0.1) differential expression at the allelic level were first detected in a pairwise manner among DVS, T19, and T78. Then those significantly upregulated or downregulated genes compared to both DVS and T78 (T19) were identified as differentially expressed genes in T19 (T78). We carried out miRNA-seq analysis using the nf-core smRNASeq pipeline v1.1.0 [103].

We mapped all RNA-seq data to DVS using HISAT2 v2.2.1. The uniquely mapped reads were counted in 100 bp continuous non-overlapping windows across the DVS genome using deepTools v3.5.0 [104] with the CPM normalization method. The read abundance in regions of interest was visualized using the Integrative Genomics Viewer v2.8.0 [105].

### Analysis of allele-specific expression and allele-expression ratio alteration

For all sweet orange transcriptomes, including the 740 downloaded from NCBI (Supplementary Table 10) and DVS, T19, and T78 RNA-seq data, the TPM (transcripts per million) normalized expression quantity of gene alleles were output using Salmon v1.4.0. Low-expression ortholog groups with <50 RNA-seq reads were filtered in each transcriptome. The allelic expression ratio of both alleles of each bi-allelic gene was calculated by dividing the allelic TPM value by the gene total TPM. Significantly biased allelic expression was inferred on a gene if the two alleles had significantly different (FDR < 0.05) mean TPM by the two-tailed t-test. To detect allelic expression ratio alteration among the accessions, we compared the DVS_A allele expression ratios, calculated as [DVS_A allele TPM / (DVS_A allele TPM + DVS_B allele TPM)], on each gene locus among DVS, T19, and T78. If the FDR was <0.05 by the two-tailed t-test, significant allelic expression ratio alteration was inferred between the two compared transcriptome groups. Hierarchical clustering and t-SNE visualization on the AEPs were carried out using python with the packages seaborn v0.11.2 and scikit-learn v1.0.2.

### qPCR quantification of gene expression

Twenty-three DEGs in T19 (Supplementary Tables 28 and 29) identified by RNA-seq were selected for quantitative real-time PCR (qPCR) verification. First-strand cDNA was synthesized from 0.3 μg of total RNA using the Affinityscript qPCR cDNA Synthesis Kit (Agilent Technologies, Santa Clara, CA, US). qPCR was performed using the Brilliant III Ultra-Fast SYBR Green QPCR Master Mix (Agilent Technologies, Santa Clara, US) following its instructions. With 18S rRNA as the reference gene [106], the 2^-ΔΔCt^ Ct method [107] was applied to analyze the qRT-PCR results.

## Acknowledgments

This work was supported in part by the United States Department of Agriculture National Institute of Food and Agriculture (NIFA) under Grant 2017-70016-26051 to F. L. and F. G., and the U.S. National Science Foundation (NSF) under Grant ABI-1759856 and MTM2-2025541 to F.L. This research was partially supported by grants from the Citrus Research and Development Foundation (CRDF 15-010, CRDF RMC 18-010, and CRDF RMC 18-011), and the New Varieties Development and Management Corporation to F.G.

## Author contributions

F.G. and F.L. conceived and designed this project. F.G. and Q.B.Y. observed and assessed the HLB tolerance of the plants. Q.B.Y carried out all the biological experiments. B.W. did all the bioinformatical and statistical analyses. B.W., Q.B.Y., F.G., and F.L. wrote the paper. Z.D and Y.P.D evaluated the plant materials and the data analysis results. All authors have read and approved the final version of this paper.

## Data availability

All sequencing data generated in this study (PacBio sequencing, NGS sequencing, and RNA-seq data) have been deposited in the National Center for Biotechnology Information (NCBI) under BioProject ID PRJNA735893. The genome assembly and gene annotation of DVS have been submitted to NCBI under the GenBank assembly accessions GCA_022201045.1 (DVS_A) and GCA_022201065.1 (DVS_B). Data supporting the findings of this work are available within the paper and its Supplementary Information files. The allelic expression patterns of 749 sweet orange RNA-seq data sets (Supplementary File 1) can be accessed from https://doi.org/10.6084/m9.figshare.19424699.v2. The scripts and configuration files used in the assembly processes and the allele-aware RNAseq pipeline are accessible from https://github.com/TheLuoFengLab/DVS-assembly-and-allele-aware-RNAseq-pipeline.git.

## Conflict interests

The authors declare no competing interests.

## Supplementary Material

Web_Material_uhac247
