# Supplementary material for: A chromosome-level phased genome enabling allele-level studies in sweet orange: a case study on citrus Huanglongbing tolerance
Source: Hortic Res. 2022 Nov 3;10(1):uhac247. doi: 10.1093/hr/uhac247 (PMC9832951; doi:10.1093/hr/uhac247)
Supplement: Web_Material_uhac247 [file web_material_uhac247.zip › 08192022 Supplementary Notes and Figures.docx]

**A chromosome-level phased genome enabling allele-level studies in sweet orange: a case study on citrus Huanglongbing tolerance**

**Supplementary Notes**

**Supplementary Note 1: Whole-genome NGS data mapping rates with DVS and HSO as reference**

A 0.4% - 1.0% higher overall mapping rate, and a 2.6% - 6.8% higher properly paired mapping rate, were achieved with DVS as the reference compared with HSO on 12 SWO whole-genome NGS data (Supplementary Fig. 2C and Supplementary Table 25). Unexpectedly, a 1.2% - 25.9% higher unique mapping rate was achieved also with DVS as the reference compared with HSO.

**Supplementary Note 2: Spontaneous structural mutations between DVS and the mother plant of T19 and T78**

Forty SVs detected between DVS and both T19 and T78 are supposed to have developed before the irradiation induction. With a mandarin genome [1] and a pummelo genome [2] as outgroup controls, thirty-one of them are inferred to be somatic mutations unique to DVS, including 30 insertions and 1 inversion. To be clear, it is important to emphasize here that the Valencia orange tree from which DNA was extracted for sequencing to produce the DVS assembly is a different tree from the source Valencia orange tree (OVS) that produced the buds that were irradiated. In that sense, these SVs represent differences detected between two different clones of Valencia sweet orange. The other nine should have developed in the OVS lineage and are shared by T19 and T78, including 5 insertions, 3 tandem duplications, and 1 deletion. Most of the insertions, 26 of the 29 in DVS and 4 of 5 in OVS, are insertions of TEs, including three Mutator-like transposable elements (MULEs) and one unclassified TE (Supplementary Table 15). On chr3A, an alternative contig involving a 6,924 bp MULE insertion is a DVS-specific mutation (INS18) and occupies ~50% of local reads (Supplementary Fig. 11).

**Supplementary Note 3: Genetic origin inference of the DVS chromosomes and switch error detection in interspecific regions**

The DVS assembly was randomly separated into two sets, each including chromosomes 1 to 9. The two chromosome sets were analyzed separately. First, the mandarin and pummelo NGS data were mapped to a chromosome set by BWA v0.7.17 [3]. Small variants and genotypes of the 40 samples were called using SAMtools v1.10 [4] and BCFtools v1.10 [5]. The genotypes were filtered by requiring a minimum genotype quality of 30 and local sequencing depth not exceeding 1.5-fold of the average sequencing depth of the sample. The reference allele was denoted ‘0’, and ‘1’ denoted the alternative allele in the genotypes. The variants with only homozygous genotypes or those with < 10 samples genotyped in either species were excluded.

The distances between sample *i* and the reference were calculated in continuous 10 kb windows as:

$d_{i}= \frac{{N_{i0/1}}\times0.5 +N_{i1/1}\times1}{N_{i0/0}+N_{i0/1}+N_{i1/1}}$ (1)

The symbols in the formula: *d_i_*, the distance between sample *i* and the chromosome set in the analyzed window; *N_i0/1_*, *N_i0/0_*, and *N_i1/1_* denote the number of variants with 0/1, 0/0, and 1/1 genotypes in sample *i*. A minimum of 10 variants was required to be genotyped for a sample to be counted in a window. One largest and one smallest distance were removed from either species in each window. When ≥ 10 samples remained available in both species, a t-test was carried out, and the reference window would be assigned to the species with significantly smaller distances (*p* < 0.05 by two-tailed t-test). For windows with not enough variants genotyped in one group, the read coverages (normalized by whole-genome sequencing depth) were compared between the two species, and the reference window would be assigned to the species with significantly higher coverage (*p* < 0.05 by two-tailed t-test). The genetic origins of repetitive regions and regions with sparse variants were inferred based on their adjacent windows. A switch error was detected when P-M and M-P switches were observed simultaneously on DVS_A and DVS_B in the corresponding orthologous windows.


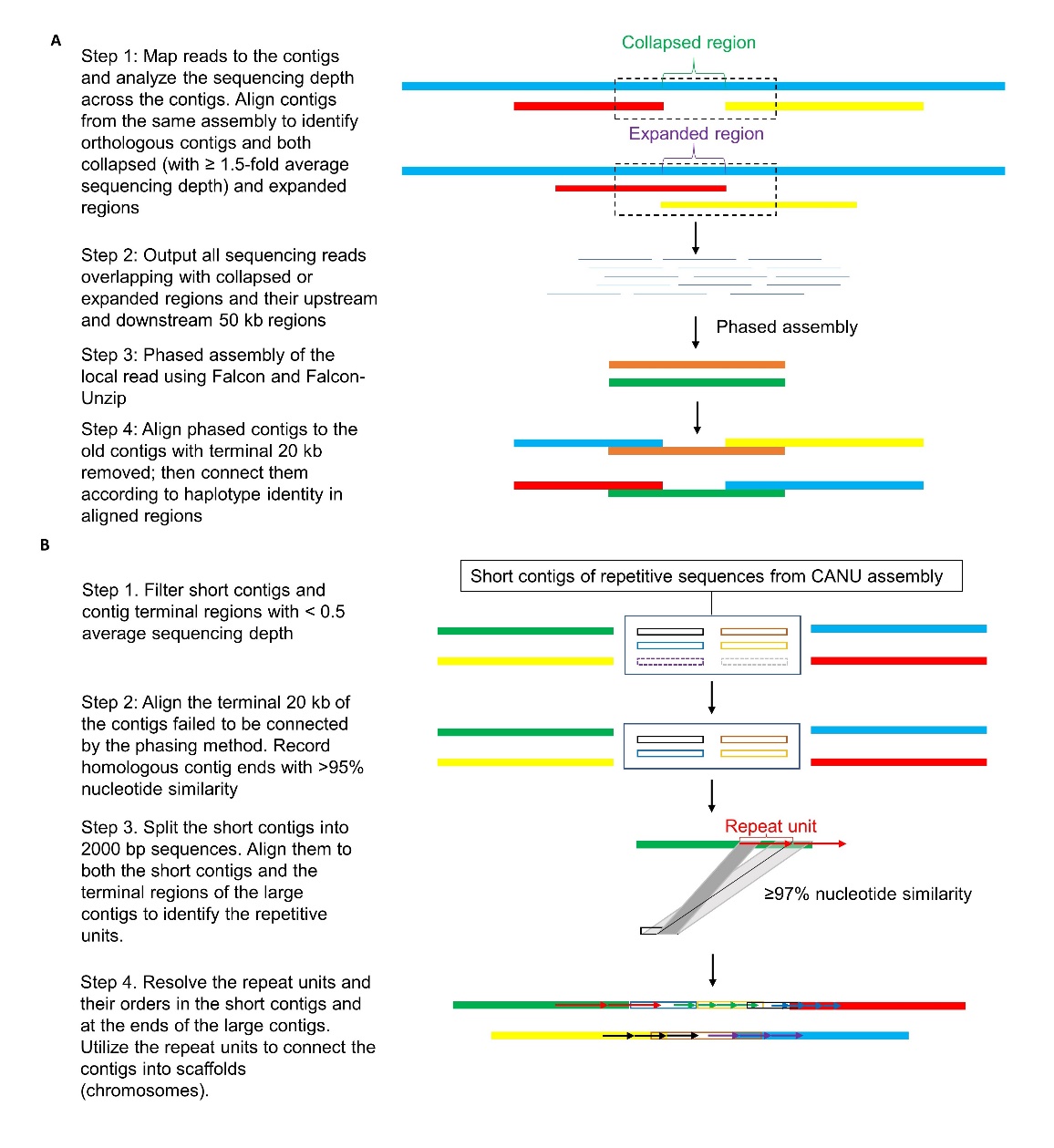


**Supplementary Figure 1.** Scaffolding the DVS contigs through phasing and repeat unit resolving. (A) Connecting contigs and fixing putative switch errors through the phased assembly of the collapsed and expanded regions. (B) Scaffolding the contigs through resolving the repeat units. CANU can separate repeats with > 3% difference [6]. Thus ≥ 97% nucleotide similarity was required to identify regions with the same repeat units. The arrows with different colors indicate different repeat units.


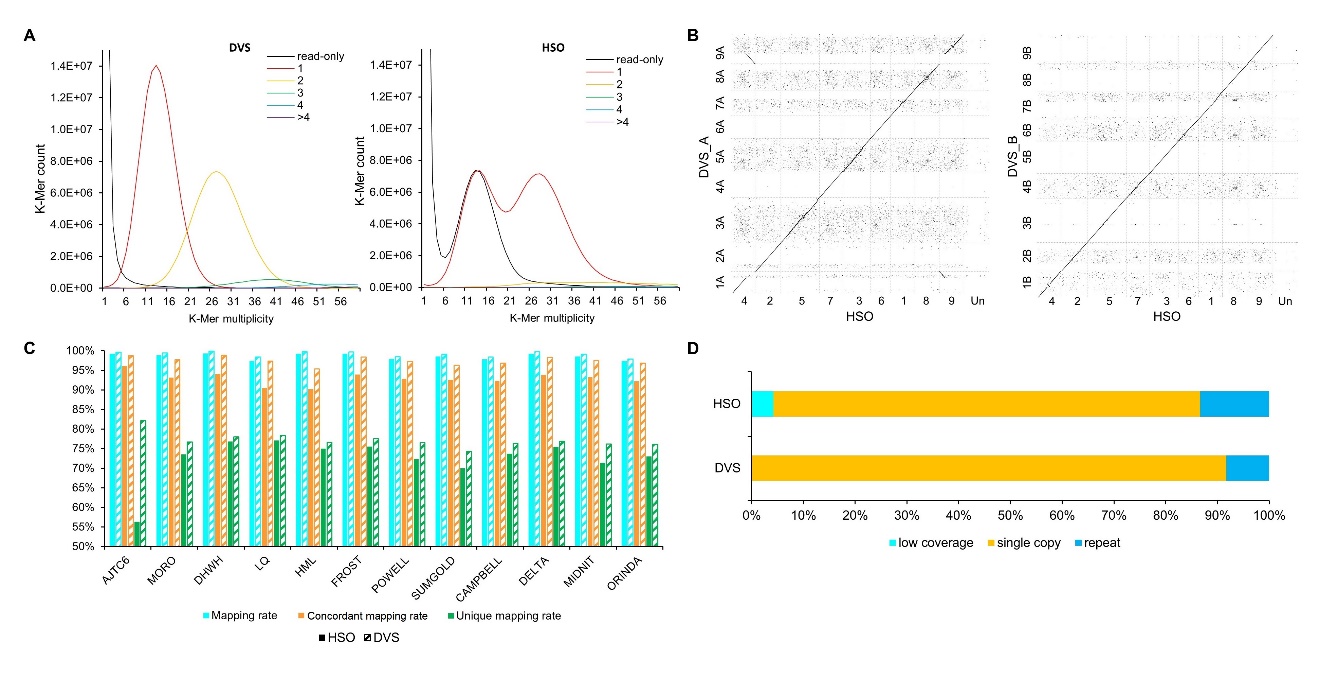


**Supplementary Figure 2.** Comparison between HSO and DVS assemblies. (A) Distribution of K-mer counts in SWO whole-genome NGS data per copy numbers found in the assemblies. The colors of the curves indicate the copy number of the K-mer in the assembly as shown in the legend. ‘read-only’ indicates the K-mer was only detected in the NGS reads. (B) Whole-genome dot-plot comparison between HSO and chromosomal sets DVS_A and DVS_B. Regions of homology are plotted as diagonal lines or dots. The D-GENIES program split the query assemblies (DVS_A and DVS_B) into ten mega-base chunks before alignment [7]. Regions aligned in large continuous fragments have fewer noisy dots. (C) Whole-genome NGS data mapping rates with HSO and DVS as the reference. Pair-end sequencing reads of 12 sweet oranges (Supplementary Table 25) were mapped to HSO and DVS with the same alignment parameters. The mapping rate is the proportion of individual reads mapped regardless of the mapping status of their paired reads. The concordant mapping rate is the ratio of reads mapped in proper pairs. The unique mapping rate is the ratio of uniquely mapped reads. (D) Proportions of low coverage (≤ 0.5-fold of sequencing depth), single copy (> 0.5-fold and < 1.5-fold), and repeated regions (≥ 1.5-fold) in HSO and DVS.


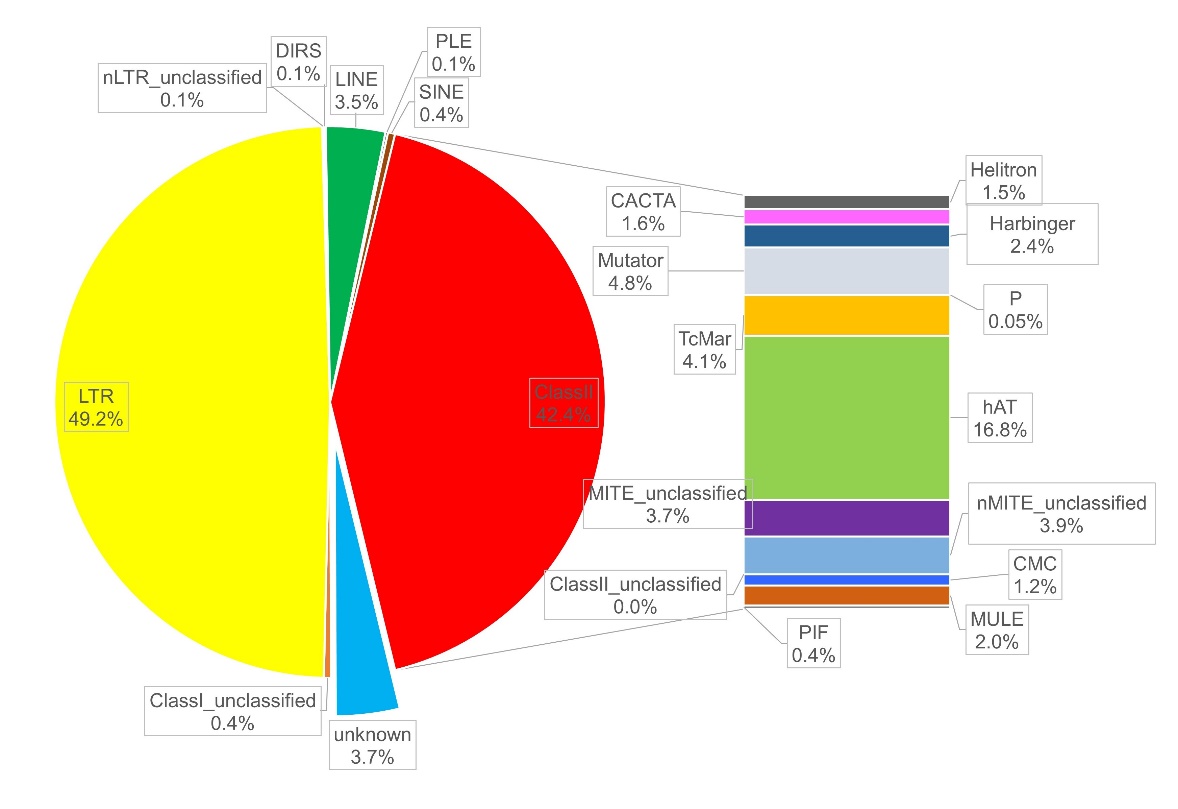


**Supplementary Figure 3.** Distribution of whole-genome TE elements in different TE types. There are 53.9% TEs classified as Class I (Retrotransposons), and 42.4% classified as Class II (DNA transposons). LTRs (152.8 Mb) account for 91.3% of Class I TEs, and LINEs (10.8 Mb) are the second most abundant Class I TEs. hAT (52.3 Mb), Mutator (15.0 Mb), and TcMar (12.9 Mb) are the most abundant Class II TE types.


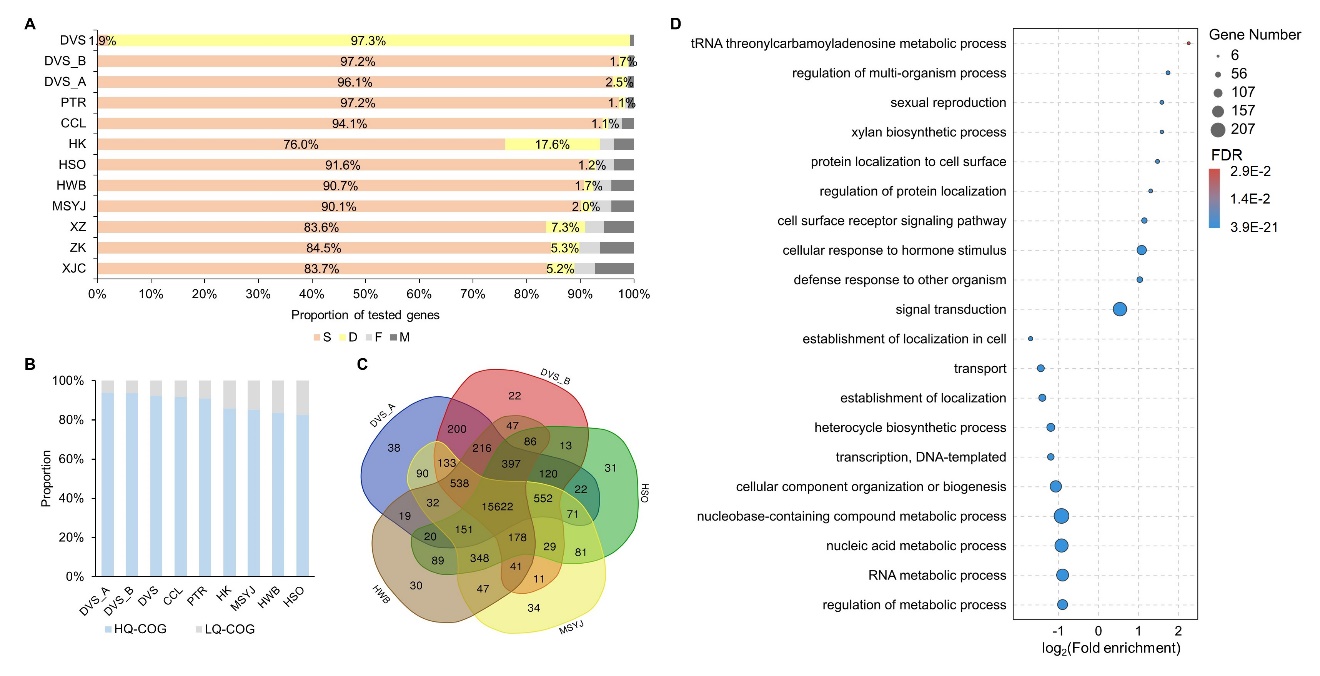
**Supplementary Figure 4.** Comparison of gene structure annotations among citrus genomes. (A) BUSCO completeness of Citrus genomes. S and D are complete genes that are single-copy and duplicated, respectively; F and M indicate fragmented and missing genes. ZK [8] and PTR [9], *Poncirus trifoliata* genomes**;** CCL [10], *Citrus clementina*; HK [11], *Citrus japonica*; HSO [12], di-haploid sweet orange v4; HWB, *Citrus maxima*; XJC, *Citrus ichangensis*; XZ [2], *Citrus medica*; MSYJ [1], *Citrus reticulata*. (B) The proportion of high-quality (HQ-COG) and low-quality (LQ-COG) orthologous groups in the citrus assemblies with the highest BUSCO completeness. (C) Venn diagram of HQ-COG sharing relationship among sweet orange assemblies and the genomes of its two parental species. (D) Overrepresented (> 0 on the horizontal axis) and underrepresented (< 0) gene ontologies (GO) in the genes without ortholog (test gene set) in DVS. Only the ten overrepresented and ten underrepresented GO terms (vertical axis) with the lowest FDR values are shown. Similar GO terms with redundancy have been manually slimmed.


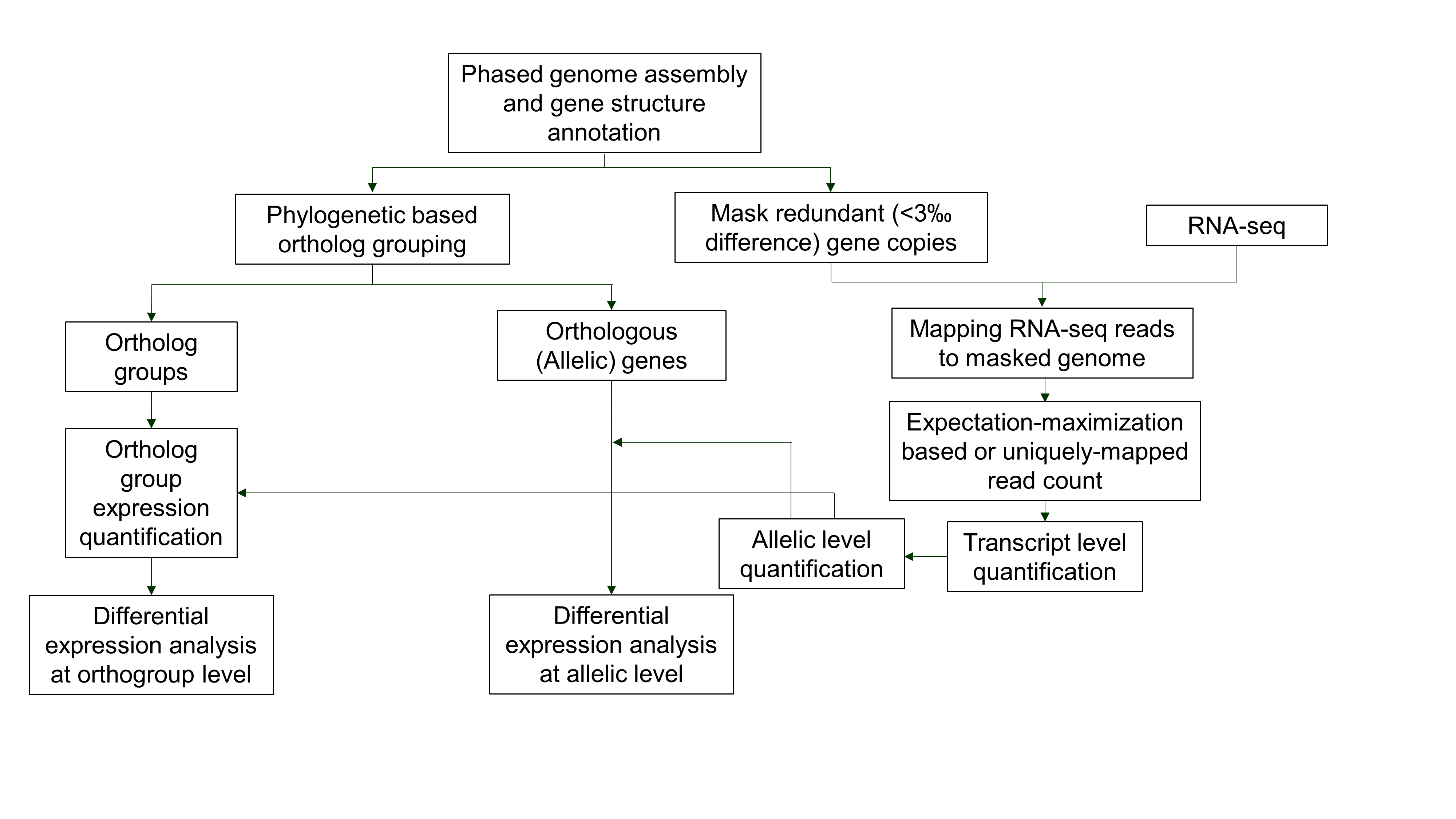


**Supplementary Figure 5.** Allele-aware RNA-seq analysis pipeline for SWO.


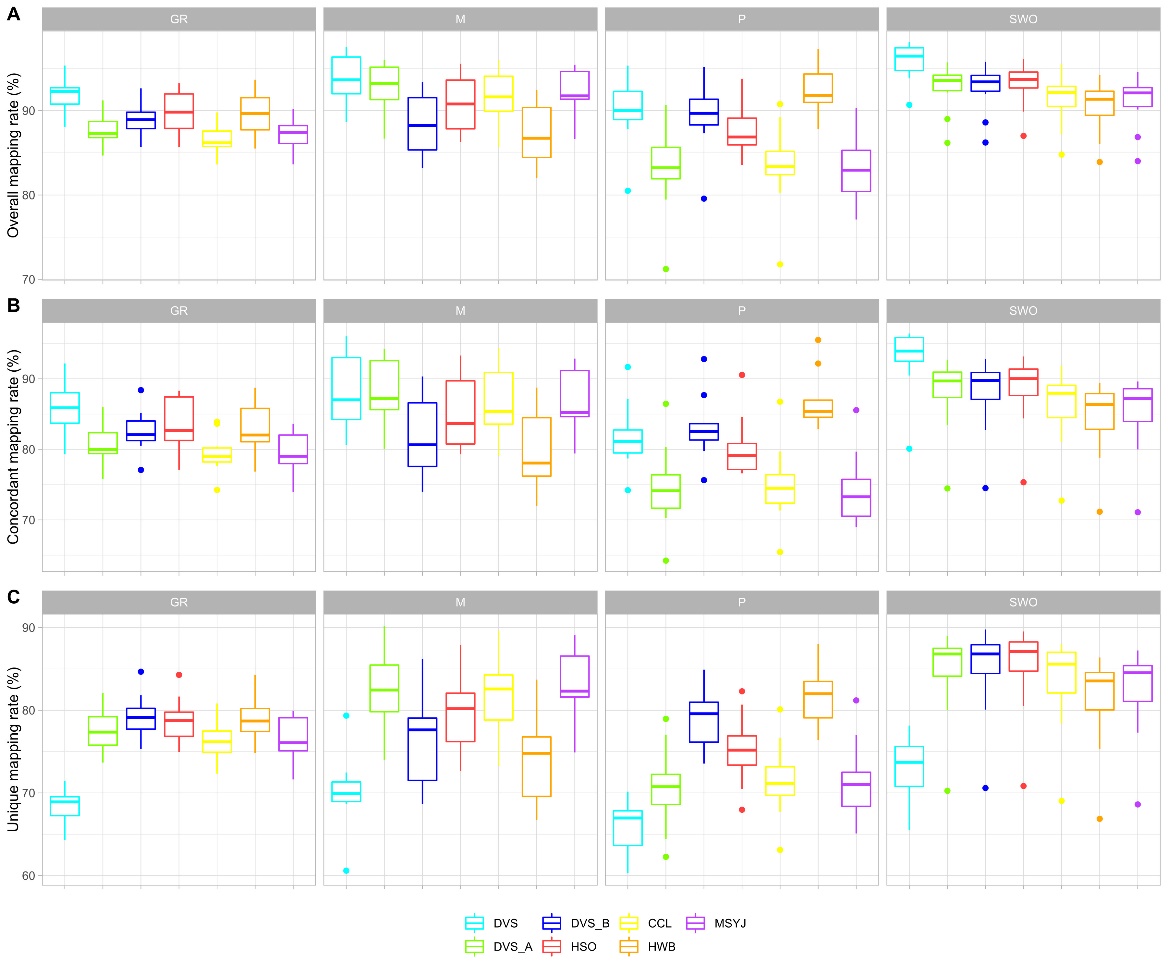


**Supplementary Figure 6.** Mapping rates of RNA-seq data with different citrus assemblies as the reference. For each of the four species, grapefruit (GR), mandarin (M), pummelo (P), and SWO, ten RNA-seq data were mapped to all the tested citrus assemblies. The boxplots describe the distribution of overall mapping rates (A), concordant mapping rates (B), and unique mapping rates (C). The overall, concordant, and unique mapping rates were calculated as the ratios of all mapped reads, concordantly mapped read pairs, and uniquely and concordantly mapped read pairs, respectively.


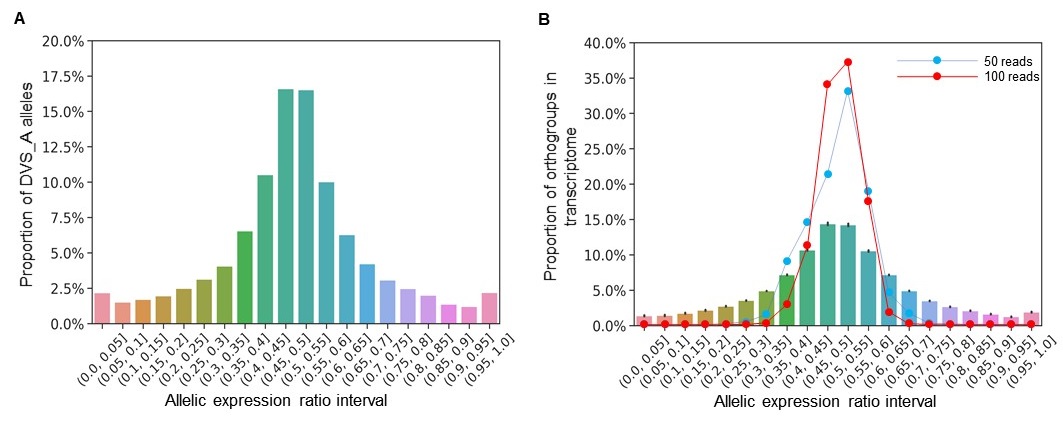


**Supplementary Figure 7.** Allelic expression ratio distribution in 740 sweet orange transcriptomes. (A) Histogram showing the distribution of the average allelic expression ratios of the DVS_A alleles in the 740 transcriptomes. The DVS_A alleles from 16,143 bi-allelic orthogroups with no less than 50 reads in at least one transcriptome were summarized in this graph. (B) Histogram showing the allelic expression ratio distributions in the 740 sweet orange transcriptomes. The distribution of the bi-allelic orthogroups was analyzed in each transcriptome, and only orthogroups with ≥ 50 available reads were counted. The histogram shows the average interval frequencies of all the transcriptomes, with the error bars showing the standard deviation. The blue and red dots linked by lines denote the distribution of the expected allelic read ratios assuming all bi-allelic orthogroups have the given read counts shown in the legend and all alleles have equal allelic expression ratios.


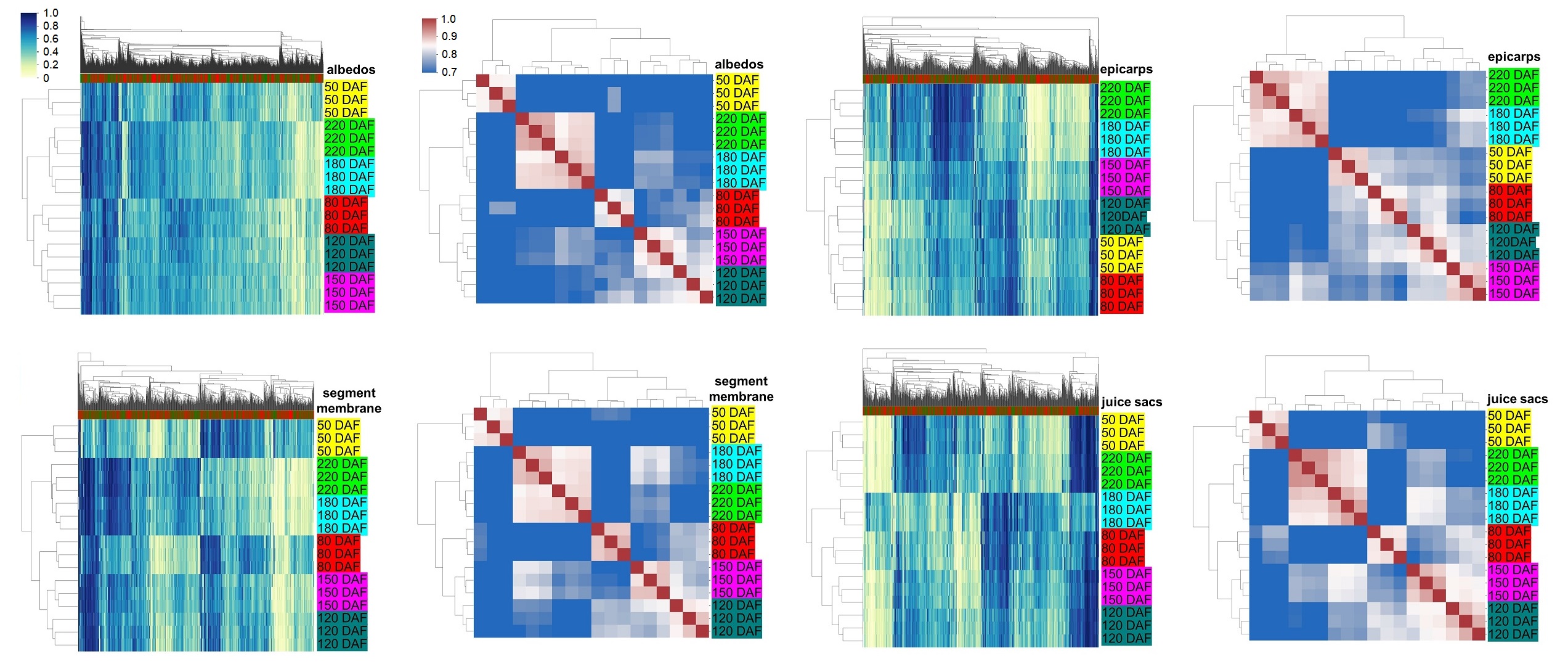


**Supplementary Figure 8.** Heatmap and hierarchical clustering of transcriptomic allelic expression patterns (AEPs) from BioProject PRJNA517400. For each tissue type: left panels, hierarchical clustering (dendrograms) and heat maps based on 500 genes with the highest expression ratio variance among the transcriptomes; each column represents a gene allele, and the colored bar on top of the heat maps indicates the alleles from DVS_A (red-colored) and DVS_B (green-colored), respectively; right panels, hierarchical clustering and heat map of AEP correlation coefficients based on 7,500 genes with the highest expression ratio variance among the transcriptomes. All the other left and right panels share the same color scales as in the albedo graphs. DAF, days after flowering.


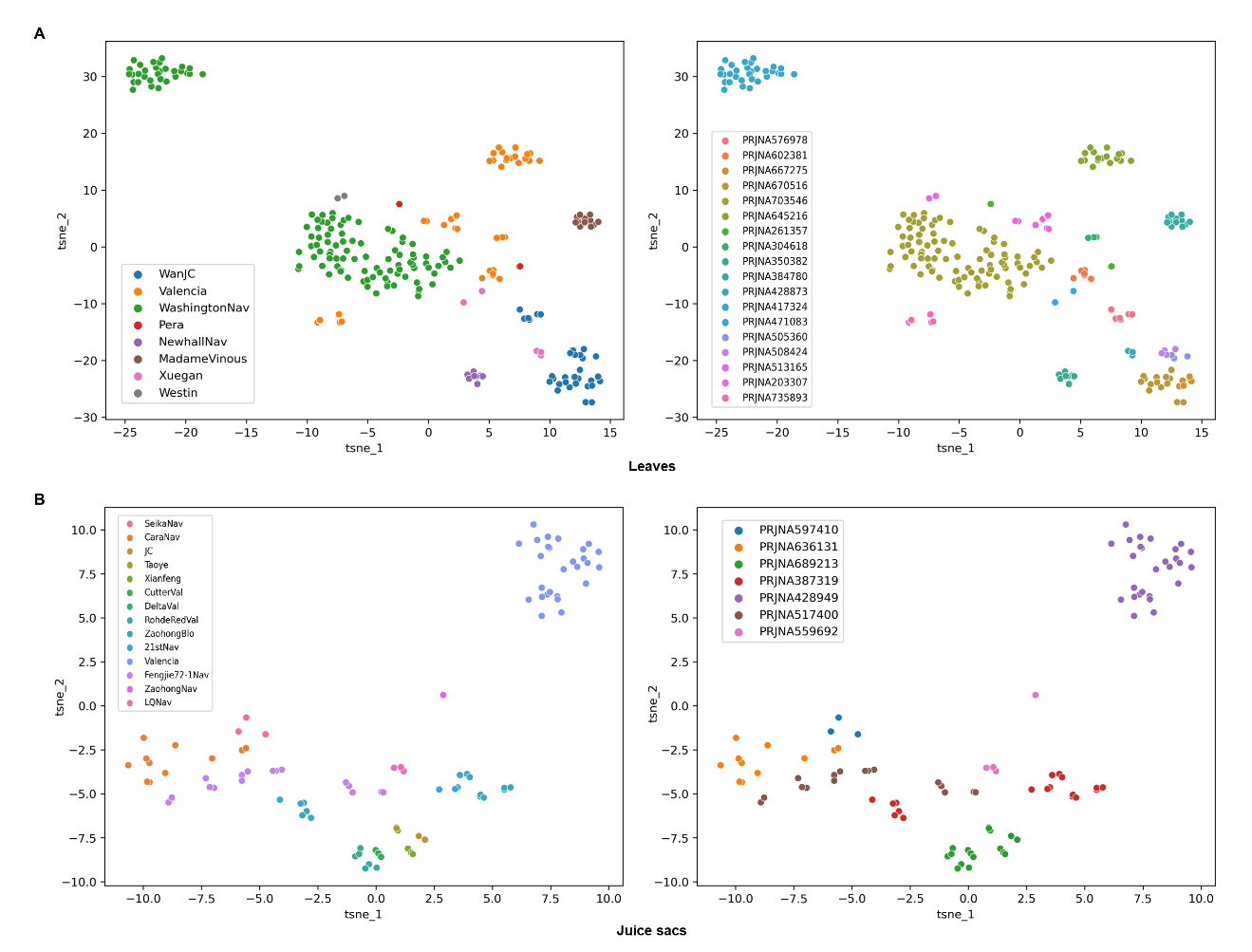


**Supplementary Figure 9.** T-Distributed Stochastic Neighbor Embedding (t-SNE) of allelic expression patterns (AEPs) of sweet orange transcriptomes. T-SNE visualization of the AEPs of the sweet orange leaf (A) and juice sac (B) transcriptomes from different cultivars (left panels) and different NCBI BioProjects (right panels). The same transcriptomes are used in the corresponding left and right panels.


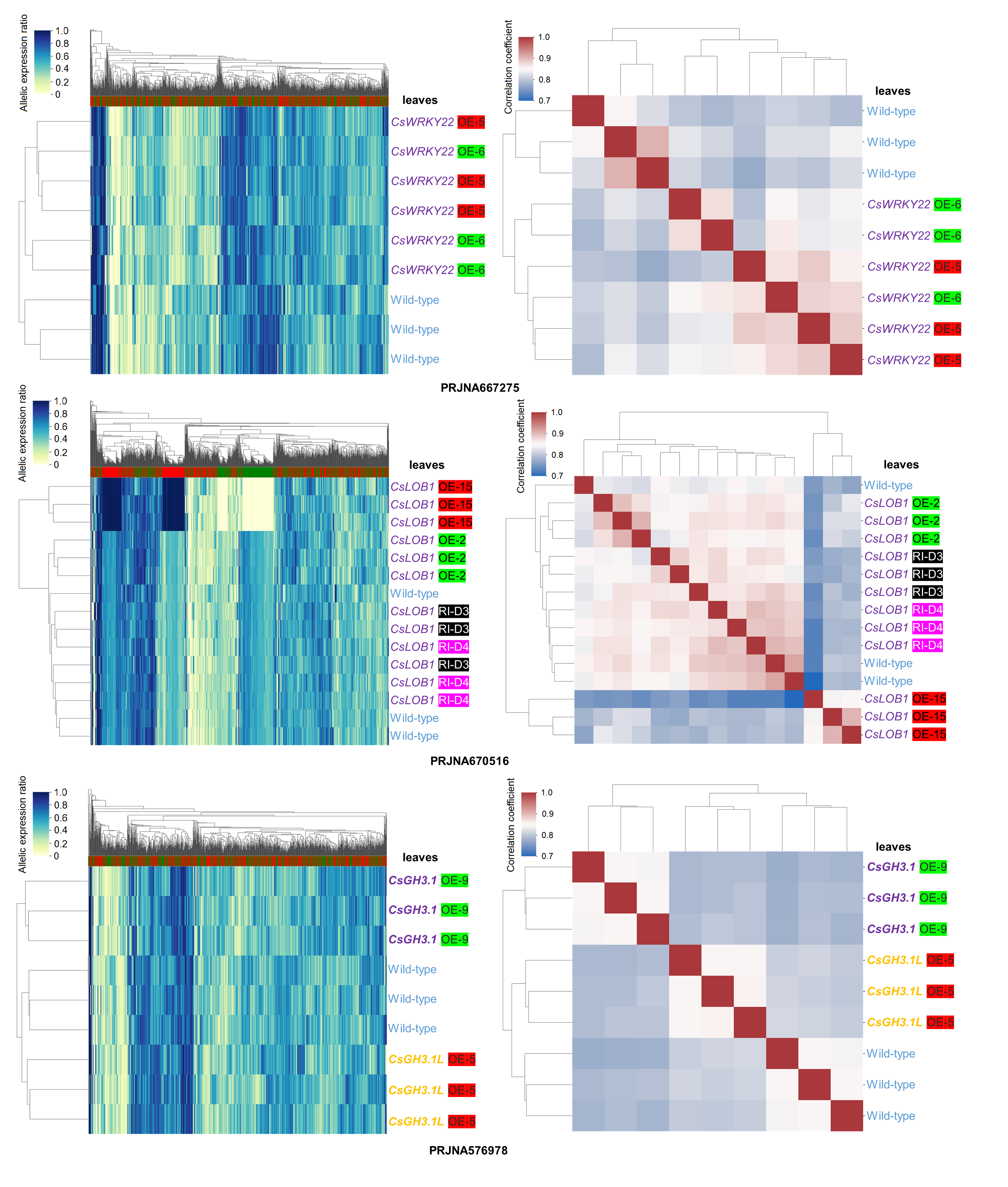


**Supplementary Figure 10**. Heatmap and hierarchical clustering of allelic expression patterns of transcriptomes from different transgenic sweet orange lineages and the non-transgenic controls. The graphs were depicted using the same method as described in the legend of Supplementary Figure 9. The column attributes include the names of the manipulated genes and the overexpression (OE-) or RNA interference (RI-) lineages. Sweet orange cultivar Wanjingcheng has been applied as the experimental plant in all three studies. The detailed information of the transgenic plants can be accessed from the corresponding studies of PRJNA667275 [13], PRJNA670516 [14], and PRJNA576978 [15].


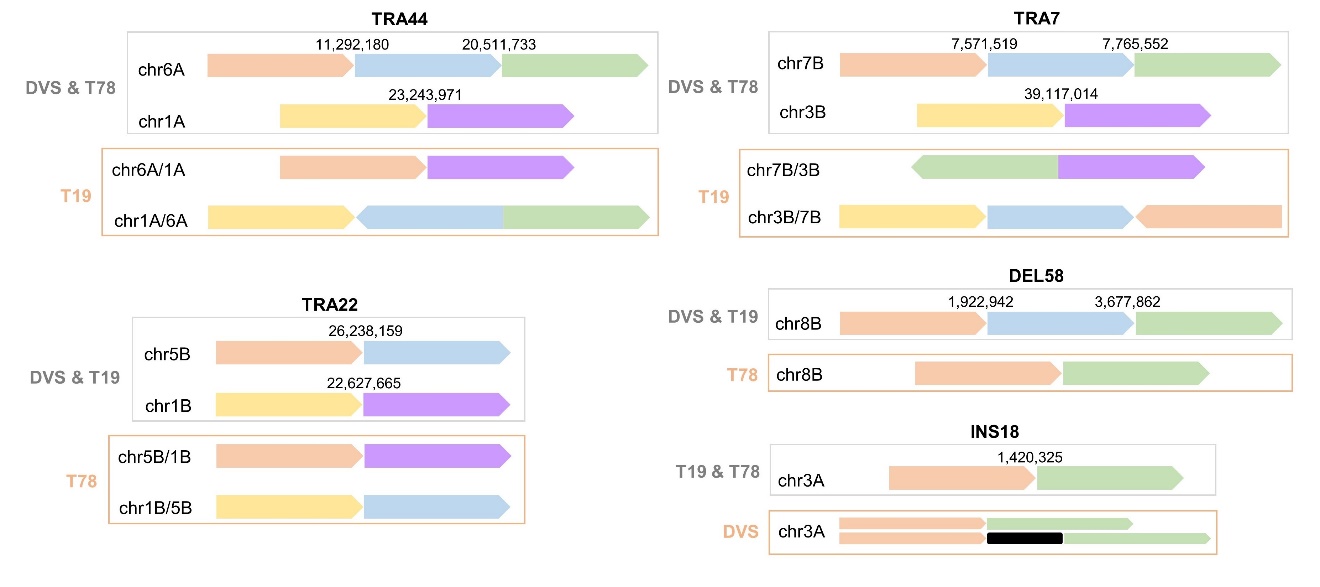


**Supplementary Figure 11.** Diagrams of five somatic structural mutations detected in T19, T78, and DVS. The arrows denote the directions from 5’ to 3’ on the DVS chromosomes. The gray font and frame indicate the ‘wild-type’ genotype, and the light red font and frame indicate the mutant accession and genotype of the corresponding mutation, respectively. The black bar in DVS of INS18 represents the 6,924 bp Mutator transposon. The wild type and the mutant type chr3A coexist with approximately 1:1 ratio in the chimeric DVS on INS18.


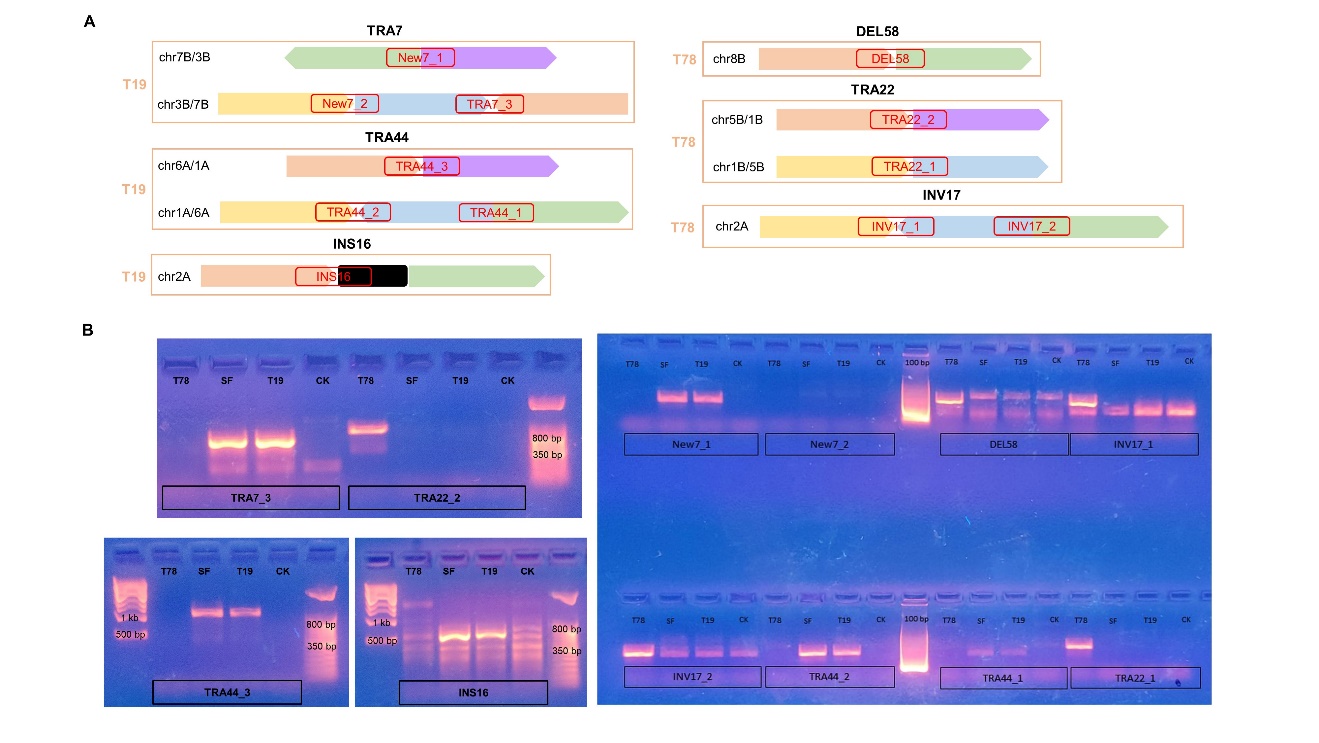


**Supplementary Figure 12.** PCR verification of 6 putative irradiation-induced structural mutations. (A) Diagrams showing the breaking end connections (connections between arrows or squares of different colors) in the mutant accession and the corresponding amplicons (red frames) designed to verify them. The arrows in panel A denote the directions from 5’ to 3’ on the DVS chromosomes. Only the mutant genotypes (the orange frames) and accession names are shown in the graph. (B) PCR amplification of the designed amplicons. CK denotes the ordinary Valencia sweet orange DVS. The primers for DEL58, INV17_1, INV17_2, and INS16 had non-special amplifications in the non-mutant accessions, but the target amplicons were only amplified with high efficiency in the corresponding mutant accession(s).


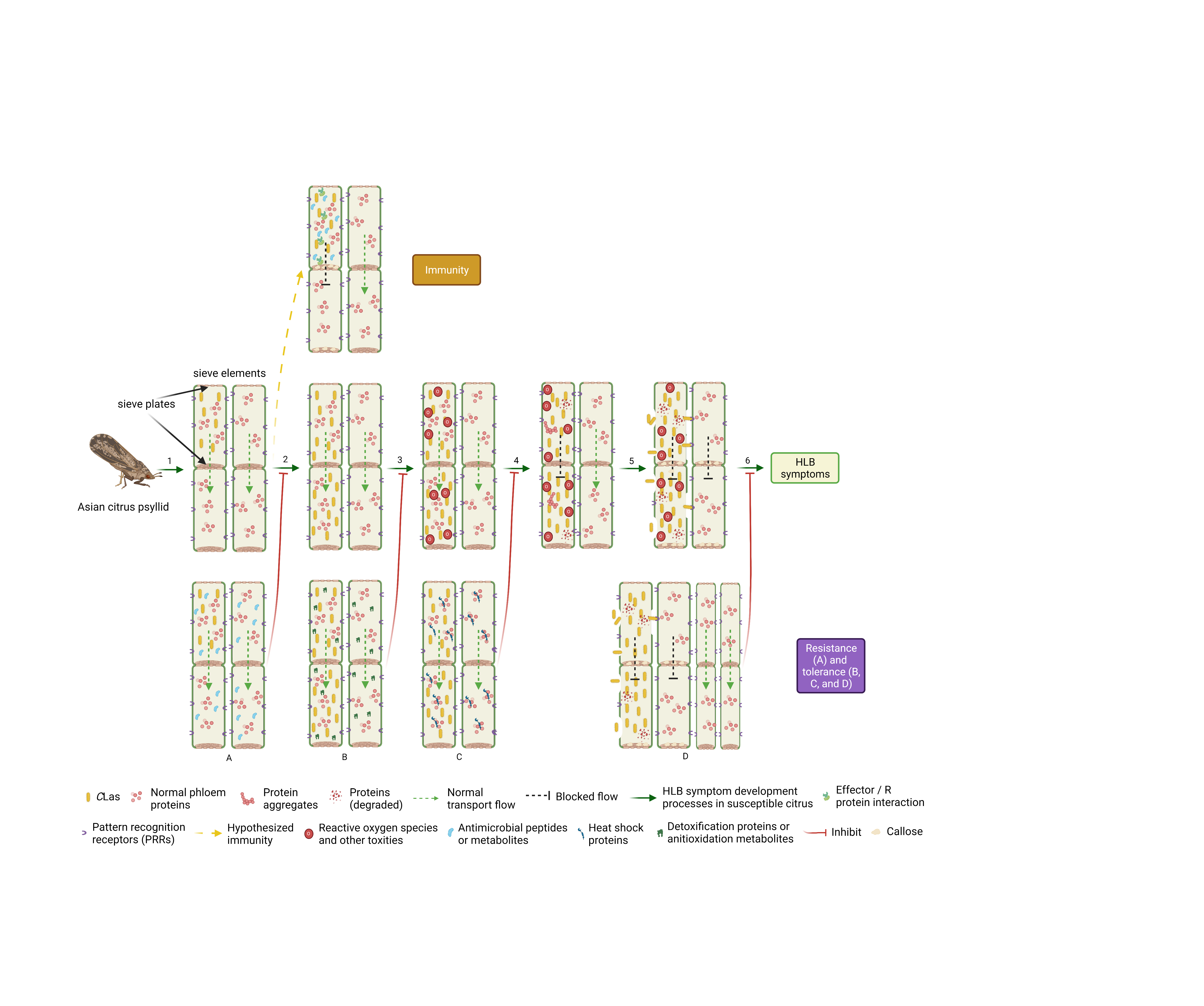


**Supplementary Figure 13.** Citrus Huanglongbing (HLB) symptom development in susceptible citrus and the mechanisms of HLB tolerance and resistance. Steps 1-6 denote the HLB symptom development process in susceptible citrus genotypes. 1, The pathogens are injected by Asian Citrus Psyllid (*Diaphorina citri* Kuwayama) into the phloem [16]; 2, *C*Las propagates in the phloem; 3, The multiplication of *C*Las in the phloem induces multiple stresses including reactive oxygen species (ROS) accumulation, reduced ATP levels, and et al.; 4, ROS and other stresses result in protein misfolding, aggregation, and degradation; 5, High content of insoluble denatured proteins (disrupted protein homeostasis) paralyzes the phloem function and induces phloem necrosis, and the microbe-associated molecular patterns of released *C*Las induce defense responses in nearby cells [17], including callose deposition and programmed cell death; 6, High rate of dysfunctional phloem causes HLB symptoms including blotchy mottles, yellow branches, and eventual tree dieback [16]. A, B, C, and D are (hypothesized) mechanisms of HLB resistance (A) and tolerance (B, C, and D) in citrus. A, Enhanced systemic acquired resistance [18-21]; B, Reliving the stresses from *C*Las multiplication by high-level of antioxidant metabolites or enhanced detoxication pathways [22-25]; C, Promoted phloem protein homeostasis due to upregulation of heat shock proteins (HSPs), which enables further induction of stress responses; D, Increased functional phloem ratio from novel high phloem regeneration rate [26,27]. The mechanism C has been proposed in this study in the HLB-tolerant T19.

**References**

1. Wang L, He F, Huang Y et al. Genome of wild mandarin and domestication history of mandarin. *Mol Plant.* 2018;11:1024–1037.

2. Wang X, Xu Y, Zhang S et al. Genomic analyses of primitive, wild and cultivated citrus provide insights into asexual reproduction. *Nat Genet.* 2017;49:765–772.

3. Li H. Aligning sequence reads, clone sequences and assembly contigs with BWA-MEM. *arXiv preprint.* 2013;arXiv:1303.3997v2.

4. Li H, Handsaker B, Wysoker A et al. The Sequence Alignment/Map format and SAMtools. *Bioinformatics.* 2009;25:2078-2079.

5. Li H. A statistical framework for SNP calling, mutation discovery, association mapping and population genetical parameter estimation from sequencing data. *Bioinformatics.* 2011;27:2987–2993.

6. Koren S, Walenz BP, Berlin K et al. Canu: scalable and accurate long-read assembly via adaptive k-mer weighting and repeat separation. *Genome Res.* 2017;27:722–736.

7. Cabanettes F, Klopp C. D-GENIES: dot plot large genomes in an interactive, efficient and simple way. *PeerJ.* 2018;6:e4958.

8. Huang Y, Xu Y, Jiang X et al. Genome of a citrus rootstock and global DNA demethylation caused by heterografting. *Hort Res.* 2021;8:69.

9. Peng Z, Bredeson JV, Wu GA et al. A chromosome-scale reference genome of trifoliate orange (*Poncirus trifoliata*) provides insights into disease resistance, cold tolerance and genome evolution in *Citrus*. *The Plant journal : for cell and molecular biology.* 2020;104:1215–1232.

10. Wu GA, Prochnik S, Jenkins J et al. Sequencing of diverse mandarin, pummelo and orange genomes reveals complex history of admixture during citrus domestication. *Nat Biotechnol.* 2014;32:656–662.

11. Zhu C, Zheng X, Huang Y et al. Genome sequencing and CRISPR/Cas9 gene editing of an early flowering Mini-Citrus (*Fortunella hindsii*). *Plant Biotechnol J.* 2019;17:2199–2210.

12. Wang L, Huang Y, Liu Z et al. Somatic variations led to the selection of acidic and acidless orange cultivars. *Nat Plants.* 2021;7:954–965.

13. Long Q, Du M, Long J et al. Transcription factor WRKY22 regulates canker susceptibility in sweet orange (*Citrus sinensis* Osbeck) by enhancing cell enlargement and CsLOB1 expression. *Hort Res.* 2021;8.

14. Zou X, Du M, Liu Y et al. CsLOB1 regulates susceptibility to citrus canker through promoting cell proliferation in citrus. *The Plant Journal.* 2021;106:1039-1057.

15. Zou X, Long J, Zhao K et al. Overexpressing GH3.1 and GH3.1L reduces susceptibility to *Xanthomonas citri* subsp. citri by repressing auxin signaling in citrus (*Citrus sinensis* Osbeck). *PLoS One.* 2019;14:e0220017.

16. Bové JM. Huanglongbing: a destructive, newly-emerging, century-old disease of citrus. *J Plant Pathol.* 2006;88:7–37.

17. Shi Q, Febres VJ, Zhang S et al. Identification of gene candidates associated with Huanglongbing tolerance, using '*candidatus* Liberibacter asiaticus' flagellin 22 as a proxy to challenge citrus. *Molecular Plant-Microbe Interactions.* 2018;31:200–211.

18. Dutt M, Barthe G, Irey M et al. Transgenic citrus expressing an *Arabidopsis* NPR1 gene exhibit enhanced resistance against Huanglongbing (HLB; Citrus Greening). *PLoS One.* 2015;10:e0137134.

19. Peng A, Zou X, He Y et al. Overexpressing a NPR1-like gene from *Citrus paradisi* enhanced Huanglongbing resistance in *C. sinensis*. *Plant Cell Rep.* 2021;40:529–541.

20. Hao G, Stover E, Gupta G. Overexpression of a modified plant thionin enhances disease resistance to citrus canker and Huanglongbing (HLB). *Front Plant Sci.* 2016;7:1078.

21. Huang C-Y, Araujo K, Sánchez JN et al. A stable antimicrobial peptide with dual functions of treating and preventing citrus Huanglongbing. *Proc Natl Acad Sci USA.* 2021;118.

22. Hijaz F, Al-Rimawi F, Manthey JA et al. Phenolics, flavonoids and antioxidant capacities in *Citrus* species with different degree of tolerance to Huanglongbing. *Plant signaling & behavior.* 2020;15:1752447.

23. Ma W, Pang Z, Huang X et al. Citrus Huanglongbing is a pathogen-triggered immune disease that can be mitigated with antioxidants and gibberellin. *Nature Communications.* 2022;13:529.

24. Balan B, Ibáñez AM, Dandekar AM et al. Identifying Host Molecular Features Strongly Linked With Responses to Huanglongbing Disease in Citrus Leaves. *Front Plant Sci.* 2018;9:277.

25. Martinelli F, Reagan RL, Dolan D et al. Proteomic analysis highlights the role of detoxification pathways in increased tolerance to Huanglongbing disease. *BMC Plant Biol.* 2016;16:167.

26. Deng H, Achor D, Exteberria E et al. Phloem regeneration Is a mechanism for Huanglongbing-tolerance of "Bearss" lemon and "LB8-9" Sugar Belle® mandarin. *Front Plant Sci.* 2019;10:277.

27. Fan J, Chen C, Achor DS et al. Differential anatomical responses of tolerant and susceptible *Citrus* species to the infection of ‘*Candidatus* Liberibacter asiaticus’. *Physiol Mol Plant Pathol.* 2013;83:69–74.
